# Specificity Matters: Unpacking Impact Pathways of Individual Interventions within Bundled Packages Helps Interpret the Limited Impacts of a Maternal Nutrition Intervention in India

**DOI:** 10.1093/jn/nxab390

**Published:** 2021-11-18

**Authors:** Shivani Kachwaha, Phuong H Nguyen, Lan Mai Tran, Rasmi Avula, Melissa F Young, Sebanti Ghosh, Thomas Forissier, Jessica Escobar-Alegria, Praveen Kumar Sharma, Edward A Frongillo, Purnima Menon

**Affiliations:** International Food Policy Research Institute (IFPRI), Washington, DC, USA; International Food Policy Research Institute (IFPRI), Washington, DC, USA; Emory University, Atlanta, GA, USA; International Food Policy Research Institute (IFPRI), Washington, DC, USA; Emory University, Atlanta, GA, USA; FHI Solutions, Washington, DC, USA; FHI Solutions, Washington, DC, USA; FHI Solutions, Washington, DC, USA; FHI Solutions, Washington, DC, USA; University of South Carolina, Columbia, SC, USA; International Food Policy Research Institute (IFPRI), Washington, DC, USA

**Keywords:** maternal nutrition, micronutrient supplementation, diet diversity, weight-gain monitoring, systems strengthening, service delivery, counseling, India

## Abstract

**Background:**

To address gaps in coverage and quality of nutrition services, Alive & Thrive (A&T) strengthened the delivery of maternal nutrition interventions through government antenatal care (ANC) services in Uttar Pradesh, India. The impact evaluation of the A&T interventions compared intensive ANC (I-ANC) with standard ANC (S-ANC) areas and found modest impacts on micronutrient supplementation, dietary diversity, and weight-gain monitoring.

**Objectives:**

This study examined intervention-specific program impact pathways (PIPs) and identified reasons for limited impacts of the A&T maternal nutrition intervention package.

**Methods:**

We used mixed methods: frontline worker (FLW) surveys (*n* = ∼500), counseling observations (*n* = 407), and qualitative in-depth interviews with FLWs, supervisors, and block-level staff (*n* = 59). We assessed 7 PIP domains: training and materials, knowledge, supportive supervision, supply chains, data use, service delivery, and counseling.

**Results:**

Exposure to training improved in both I-ANC and S-ANC areas with more job aids used in I-ANC compared with S-ANC (90% compared with 70%), but gaps remained for training content and refresher trainings. FLWs’ knowledge improvement was higher in I-ANC than S-ANC (22–36 percentage points), but knowledge of micronutrient supplement benefits and recommended foods was insufficient (<50%). Most FLWs received supervision (>90%), but supportive supervision was limited by staff vacancies and competing work priorities. Supplies of iron–folic acid and calcium supplements were low in both areas (30–50% stock-outs). Use of monitoring data during review meetings was higher in I-ANC than S-ANC (52% compared with 36%) but was constrained by time, understanding, and data quality. Service provision improved in both I-ANC and S-ANC areas, but counseling on supplement benefits and weight-gain monitoring was low (30–40%).

**Conclusions:**

Systems-strengthening efforts improved maternal nutrition interventions in ANC, but gaps remained. Taking an intervention-specific perspective to the PIP analysis in this package of services was critical to understand how common and specific barriers influenced overall program impact.

## Introduction

Maternal undernutrition is a longstanding and significant public health concern (1) contributing to maternal mortality, fetal growth restriction, neonatal deaths, and child undernutrition (2). Nearly 3.1 million annual child deaths and 35% of the disease burden among children <5 y are attributable to maternal undernutrition globally (2, 3). These consequences are exacerbated in low- and middle-income countries (LMICs), particularly South Asia, which accounts for a third of global undernutrition (3). In India, maternal and child undernutrition is a predominant risk factor for 68% of under-5 child deaths (4), with a third of women having low BMI, more than half of pregnant and non-pregnant women having anemia, and 19% of babies born with low birth weight, of whom 4.4 million have intrauterine growth retardation (5). Improving maternal nutrition is critical for survival, health, human development, and protecting future generations (1, 6).

High coverage and quality of nutrition-specific interventions through antenatal care (ANC) platforms are essential for improving maternal nutrition. A recent study in 81 LMICs estimated a reduction of 28% in maternal and neonatal deaths and 22% fewer stillbirths if evidence-based nutrition interventions are delivered to mothers already seeking care (7). Although global momentum on the importance of nutrition interventions has increased, the coverage of most interventions falls below the reach of health services through which they are delivered (8). There are concerns especially about the effectiveness and quality of maternal nutrition interventions, including counseling and micronutrient supplementation in LMICs and South Asia (9–11).

To address the high burden of maternal undernutrition (5) and critical opportunity gaps between delivery platforms and corresponding nutrition interventions (12), the Government of India prioritized provision of essential nutrition interventions through the ANC platform (13, 14). Maternal nutrition interventions in India are delivered through 2 national programs: the Integrated Child Development Services (ICDS) managed by the Ministry of Women and Child Development and the Reproductive Maternal Newborn Child and Adolescent Health program under National Health Mission by the Ministry of Health and Family Welfare. Despite programmatic efforts, coverage and quality of maternal nutrition interventions remain suboptimal, and knowledge gaps exist on how to strengthen the existing ANC platform.

Renewed commitment to and insights from implementation research on interventions targeting the first 1000 days are urgent priorities for progress to be made in improving maternal and child undernutrition (15). Alive & Thrive (A&T) aimed to strengthen delivery of interpersonal counseling, micronutrient supplements, and community mobilization by integrating a package of maternal nutrition interventions within the government ANC platform in Uttar Pradesh (16). The impact evaluation of the A&T maternal nutrition interventions (2017–2019) found substantial secular improvements in service coverage but limited intervention impacts on micronutrient supplementation, diet diversity, and gestational weight gain (8–12 percentage point improvement) (16). Beyond traditional evaluation methods that conducted impact evaluations using only quantitative methods (17), additional methods to assess how or why impacts are achieved or not achieved are emerging (18). Previous studies have used a program impact pathway (PIP) analysis to examine program fidelity and identify critical steps in the implementation of behavior change communication strategies to improve infant and young child feeding in Bangladesh (19), Ethiopia (20), and Vietnam (21). To our knowledge, no study, however, has conducted a PIP analysis of multiple maternal nutrition interventions, including micronutrient supplementation, weight-gain monitoring, and diet counseling bundled into a package. In this implementation-science study, we examined intervention-specific impact pathways to identify reasons for limited impacts of the A&T overall package of interventions on maternal nutrition practices.

## Methods

### Study context and intervention description

This study used data from the impact evaluation of A&T's interventions that aimed to strengthen maternal nutrition services within the existing ANC government program in 2 districts of Uttar Pradesh (Unnao and Kanpur-Dehat) over 18 months from 2017 to 2019. The interventions included provision and counseling on iron–folic acid and calcium supplements, gestational weight-gain monitoring, interpersonal counseling on weight gain and diet during pregnancy, and community mobilization (22).

There were 2 intervention arms: *1*) intensive ANC (I-ANC) areas that received the above interventions in addition to standard government services and *2*) standard ANC (S-ANC) areas that received standard government services. A&T worked with 3 types of government frontline workers (FLWs): Anganwadi Workers (AWWs), Accredited Social Health Activists (ASHAs), and Auxiliary Nurse Midwives (ANMs); each of them has different tasks for providing maternal nutrition counseling to women through the course of their pregnancy (22). ANMs had a minimum of 4 contacts with women during the ANC checkup, with 1 contact each during first and second trimesters and 2 contacts during the third trimester. AWWs and ASHAs visited pregnant women at home for counseling. AWWs conducted a minimum of 3 visits (i.e., 1 visit during the second trimester and 2 visits during the third trimester), whereas ASHAs conducted a minimum of 4 visits (i.e., 1 visit each during the first and second trimesters and 2 visits during the third trimester).

Descriptions of the interventions, evaluation design, and main impacts on maternal nutrition practices have been published (16). Briefly, the impact evaluation design used a pair-matched clustered randomized design using block as the unit of randomization. Thirteen pairs were formed of 26 blocks from the 2 districts based on several demographic, infrastructure, and amenity characteristics from census 2011 data. A propensity score–matching method was used to identify pairs of intervention and control blocks. One block from each pair was randomly assigned to I-ANC (13 blocks) or S-ANC (13 blocks) through manual lottery.

### Program impact pathways

**FIGURE 1:**
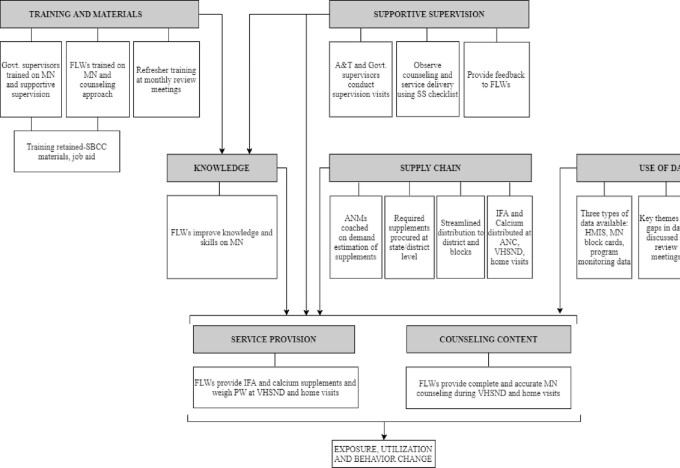
Overall program impact pathway. A&T, Alive & Thrive; ANC, antenatal care; ANM, auxiliary nurse midwife; FLW, frontline worker; HMIS, Health Management Information System; IFA, iron and folic acid; MN, maternal nutrition; PW, pregnant women; SBCC, social and behavior change communication; SS, supportive supervision; VHSND, village health, sanitation, and nutrition day.

The PIP was developed to map the mechanisms through which the A&T interventions were expected to achieve impact. The PIP was developed through an iterative process: review of program documents, close discussions with A&T staff, and multiple revisions based on feedback. The final intervention-specific PIP is presented in **Figure 1**, and details of program interventions and expectations along the PIP are in **Table 1**. For this article, we studied 7 domains related to improving maternal nutrition service delivery.

**TABLE 1:** Program interventions and expectations along program impact pathway to improve service provision^1^

PIP	Interventions	Expectations
Trainings on MN and distribution of job aid materials for FLWs and supervisors	• FLWs receive a 1-d training on MN, including information on IFA and calcium supplements, diet diversity, weight gain during pregnancy, and interaction with pregnant women and family for delivering services.• Supervisors receive 1-d training on supportive supervision principles, including overseeing effective delivery of MN services, supervision visits, and use of checklist during visits.• Refresher trainings (∼2 h) on specific topics of MN are provided during monthly meetings.• FLWs and supervisors are provided SBCC materials and job aid, including MN calendar, flipchart, and flier to retain topics of training	• Staff positions are filled, and staff are available for training.• All the required personnel attend the training.• Enough job aid and materials are available to distribute to FLWs.• FLWs can read and understand materials.• Monthly review meetings take place and refresher training is provided
Strengthening supportive supervision for FLWs	• Supervisors conduct regular visits to observe service delivery and counseling by FLWs and provide feedback using a “supportive supervision” checklist.• A&T staff accompany each FLW at least twice during home visits or VHSNDs and document activities using a supportive supervision checklist.• A&T staff and government supervisors conduct joint supervisory visits to observe service delivery by FLWs.• A&T staff provide onsite support to government supervisors for supportive supervision for FLWs	• Supervisory staff are available to conduct visits and conduct the required number of visits.• A&T staff visit FLWs and conduct joint visits with supervisors as planned.• Supportive supervision checklists are available.• A&T staff provide timely and constructive feedback to government supervisors and FLWs.• Government supervisors provide timely and constructive feedback to FLWs
Improving knowledge and skills of FLWs to deliver MN services	• FLWs’ knowledge and skills related to MN improve through exposure to training, performance of improved practices, use of job aid material, and supervision visits.• FLWs use feedback to improve MN knowledge and skills	• A 1-d training along with refresher session is provided.• FLWs and supervisors understand, value, and retain training content.• FLW and supervisors adopt improved practices.• FLW motivation improves through training and supportive supervision.• FLWs understand, value, and know how to apply feedback
Strengthening of IFA and calcium supplementation supply chain	• A&T provides technical assistance to government staff on forecasting and procurement of supplements.• A&T orients ANMs on scientific estimation of IFA and calcium supplements, proper indentation, and need-based dispensing.• ANMs make timely and accurate projections to demand for supplements from block and district levels.• State and district officials are oriented on accurate forecasting and procurement, resulting in improved procurement.• Based on correct demand generation, availability of supplements at the district and blocks.• Support provided to districts on need-based distribution of supplements to blocks.• ANM receives adequate supplements based on number of pregnant women in catchment area.• ANMs and ASHAs distribute IFA and calcium supplements to pregnant women for each month of pregnancy during home visit contacts and ANC checkups during VHSNDs	• ANM positions are filled and ANMs are available for orientation.• ANMs are capable and motivated to make timely and accurate indents following orientation.• State and district staff can forecast and procure supplements following technical assistance from A&T.• Sufficient stock of supplements is available at districts and blocks for supply.• Supply of supplements improves based on demand created.• ANMs and ASHAs distribute timely supplements to pregnant women
Strategic use of data to track the progress of interventions and identify needed improvements	• A&T and government staff hold data review meetings once a month to review MN data quality.• Government staff ask ANMs to make corrections based on errors identified in data.• A&T staff prepare a monthly block card of key MN indicators using available data.• Monthly review meetings are used to discuss data and identify key gaps and areas for improvement in service delivery	• Government staff are available and have capacity to collect, monitor, review, and make decisions on data.• ANMs have sufficient time and capacity to collect and make corrections to HMIS data.• Government staff understand, value, and are motivated to use MN block cards and adopt improved practices.• There is sufficient time for data-related discussions and decisions to take place during monthly meetings.• Data are used effectively to drive discussion on MN activities and identify areas for improvement
Intensifying MN service delivery	• AWW visits each pregnant woman at least 3 times during pregnancy (1 time during second trimester and 2 times during third trimester).	• Staff are available to provide MN services.• FLWs conduct home visits and VHSNDs as planned.• Required supplies of IFA and calcium tablets, weighing scale,
	• ASHA visits each pregnant woman at least 4 times during pregnancy (1 time each during first and second trimesters and 2 times during third trimester).• ASHA and AWW mobilize women and family members to visit VHSNDs for ANC checkups.• ANMs have a minimum of 4 contacts with pregnant women during ANC at VHSNDs (1 time each during first and second trimesters and 2 times during third trimester)	and equipment for ANC checkup are available during VHSNDs.• FLWs understand how to use equipment correctly and adopt improved practices
Improving interpersonal counseling and quality contacts during pregnancy	• FLWs provide complete and accurate MN counseling, including problem solving on IFA and calcium supplements, diet diversity, and weight gain during home visits and VHSNDs.• Individual key messaging/communication and group-based counseling is provided during VHSNDs.• FLWs use job aid for counseling	• Staff are available to provide counseling.• FLWs can provide complete and accurate counseling based on improved knowledge and skills and adopt improved practices.• Job aid and SBCC materials are available

1 A&T, Alive & Thrive; ANC, antenatal care; ANM, auxiliary nurse midwife; ASHA, accredited social health activist; AWW, Anganwadi worker; FLW, frontline worker; HMIS, Health Management Information System; IFA, iron and folic acid; MN, maternal nutrition; SBCC, social and behavior change communication; VHSND, village health, sanitation, and nutrition day.

### Study design, participants, and data collection

We used mixed methods to gather data on the 7 PIP domains: *1*) quantitative surveys with FLWs and supervisors, *2*) structured observations of counseling sessions, and *3*) qualitative in-depth interviews with FLWs, supervisors, block-level staff, and A&T program staff. A partially mixed sequential dominant status design was used with an emphasis on complementary quantitative and qualitative methods (23, 24). The quantitative data were collected first, followed by qualitative data collection, to inform quantitative findings. Data collection tools were carefully designed around the specific needs of each intervention in the bundled package.

### Frontline worker and supervisor surveys

The FLW survey was conducted with 497 FLWs in 2017 (*n* = 253 in I-ANC and 245 in S-ANC) prior to the implementation of the intervention and with 459 FLWs (*n* = 231 in I-ANC and 248 in S-ANC) in 2019 (after 2 y of intervention) using a structured questionnaire. FLWs were asked about receipt of training, job aid materials, and supportive supervision in the previous 6 months. Knowledge of micronutrient supplements was assessed using questions related to quantity, frequency, and benefits of supplements. Knowledge of diet diversity was assessed by asking FLWs how a pregnant woman should eat to provide good nutrition to her baby, including number of food groups and different recommended foods during pregnancy. Knowledge of weight gain was assessed based on FLWs’ correct response to how much weight a woman should gain during pregnancy (10–12 kg). For service delivery, FLWs were asked about frequency of service provision and counseling content related to maternal nutrition during village health, sanitation, and nutrition days (VHSNDs) and home visits to pregnant women. Supervisor interviews (*n* = 103 in I-ANC and 106 in S-ANC in 2019) focused on use of data to track progress of interventions and identifying challenges in using data.

### Counseling session observations

Observations of counseling sessions were used to assess the extent to which FLWs adhered to the standards of care and to evaluate their performance. A counseling observation checklist was developed based on the guidelines for the maternal service interventions, including key counseling messages related to iron–folic acid and calcium supplements, diet diversity, weight-gain monitoring, and counseling skills. In total, 405 FLWs (*n* = 195 in I-ANC and 210 in S-ANC) were observed during counseling sessions conducted at home visits or VHSNDs.

### Qualitative in-depth interviews

Qualitative in-depth interviews were conducted with FLWs (*n* = 24), supervisors (*n* = 12), block-level staff (*n* = 20), and A&T program staff (*n* = 3) in 4 blocks belonging to I-ANC areas using a semistructured interview guide. Interviews were focused on perspectives about and experiences of program implementation, specifically, experiences with trainings and supervision, use of data during review meetings, supply chain of iron–folic acid and calcium supplements, and challenges faced in service delivery and counseling.

All study tools were developed in English and translated into Hindi. The tools were pretested locally for cultural relevance and understanding and then modified accordingly. The surveys and in-depth interviews were conducted in Hindi by fluent enumerators, supervised by field managers and researchers. Enumerators were recruited locally and trained on technical content as well as security and confidentiality issues by mixed methods (lecture, role-play, mock interview, and practice) in a classroom and field settings. Field supervisors received additional training related to quality control processes; cross-checking, editing, and coding of the questions; and security and confidentiality issues. Qualitative interviews were recorded, and field notes were taken by researchers. Enumerators conducted interviews in a separate area with only the respondent present to ensure privacy and confidentiality. Interviews took approximately 45 min.

### Data analysis

Quantitative data were analyzed using Stata 16.0 (StataCorp LP). Descriptive statistics were used to summarize the sample characteristics. Differences between I-ANC and S-ANC areas at baseline and endline were estimated using linear (for continuous variables) or logistic regression models (for categorical variables). A difference-in-differences (DID) analysis was conducted to estimate differences in changes over time between the 2 study groups (25). All models adjusted for clustering at the village level using a robust sandwich estimator.

Qualitative audio recordings were transcribed and independently translated back into English and compared with the original as a quality check. Interviews were transcribed by 2 local research assistants and stored on a password-protected computer to ensure privacy. The researchers randomly selected 10% of the transcripts for comparison with the audio files for quality control. We used MaxQDA and Microsoft Excel to organize interview transcripts and to manage data for identifying and summarizing inductive and deductive themes that corresponded to each PIP domain. Thematic analysis of the qualitative data was done (26). Memos were created to keep track of thought processes, categories, and themes and to develop codes. Inductive codes were created based on interview transcripts, field notes, and memos. Deductive codes were created based on themes addressed by questions in each interview guide. Inductive and deductive codes were consolidated into separate codebooks based on respondent group. Data were analyzed by 1 team member fluent in both Hindi and English, and codes and analysis were discussed and reviewed by the principal investigator throughout. Additional information on qualitative data analysis and tools has been reported previously (27).

Successes and challenges along PIP domains were identified through triangulation of quantitative (to obtain results on magnitude and direction of changes in PIP domains) and qualitative data (to identify the emerging themes on how the implementation of PIP domains occurred). For example, for the training domain, we examined the proportion of FLWs in I-ANC and S-ANC areas who were trained in the past year as well as the percentage trained on maternal nutrition topics. We further examined data from the in-depth interviews to inform the trends observed with FLWs’ perceptions and feedback on training received. We analyzed specific successes and challenges for different PIP domains relevant to each individual intervention. Triangulation allows development of a comprehensive understanding of evidence-based pathways and maximizing trustworthiness through the convergence of information from different data sources (28).

### Ethical clearance

This study was approved by the ethical review board from the International Food Policy Research Institute, Emory University, and the Suraksha Independent Ethics Committee in India. Verbal informed consent was obtained from all participants at all stages of data collection. The study was registered at clinicaltrials.gov as NCT03378141.

## Results

### Sample characteristics

FLW characteristics were similar in I-ANC and S-ANC areas (**Supplemental Table 1**). Among 3 types of FLWs, ASHAs were younger (37 y) than AWWs and ANMs (43–44 y). Nearly half of ASHAs had lower than secondary school education, whereas most AWWs and ANMs completed secondary school or higher. Most FLWs identified as Hindus, and ∼75% belonged to disadvantaged socioeconomic groups, specifically scheduled caste/tribe.

### Training and job aids

Coverage of training was high overall at endline, but key gaps were prevalent in exposure to maternal nutrition topics during trainings (**Table 2**). Most AWWs and ASHAs in I-ANC and S-ANC areas received maternal nutrition training in the past year (>90%), whereas ANMs in I-ANC areas were more likely than those in S-ANC areas to receive training (79% compared with 46%) (**Table 3**). Although coverage of training topics for iron–folic acid and calcium improved between baseline and endline, coverage was <50% and was similar for AWWs and ASHAs in both areas. ANMs in I-ANC areas were more likely than those in S-ANC areas to be trained on iron–folic acid and calcium (52% compared with 22%). Around 70% of AWWs and ASHAs received training on diet counseling, similar in both areas. A higher proportion of ANMs in I-ANC areas received diet training than those in S-ANC areas (60% compared with 30%). Training on weight gain was received by only about a third of FLWs, similar between areas for AWWs and ASHAs, and it was higher among ANMs in I-ANC areas compared with S-ANC areas (33% compared with 8%).

**TABLE 2:** Program impact pathways for provision of maternal nutrition interventions^1^

PIP domain	Success	Challenge
Training and materials		
Exposure to training	• Most FLWs (>90%) received training on MN in the past year	• Nearly 30% vacant positions among ANMs• Inadequate refresher training
Training content	• 50–60% FLWs received training on IFA and calcium demand estimation and supplementation• ∼60–70% FLWs received training on diet; ANMs in I-ANC areas had significantly higher exposure compared with S-ANC areas (60% compared with 30%)• ANMs in I-ANC areas had significantly higher exposure to weight-gain content during trainings compared with those in S-ANC areas (33% compared with 9%)• FLWs perceived training improved their knowledge of MN• FLWs were better able to understand training content delivered through videos	• Moderate exposure to training topics related to IFA and calcium• Lower exposure to training topics related to diet among ANMs compared to AWWs and ASHAs• Low exposure to training topics related to weight gain among all FLWs (≤33%)
Access to job aid	• Most FLWs in I-ANC received MN calendar (>90%) and flipchart (60–80%)	• Gaps in receipt of other materials, particularly audio-visual job aid (<30%)
Use of job aid	• Most ASHAs and ANMs (80–90%) reported using materials during home visits or VHSNDs and found them easy to use• FLWs perceived using materials made counseling more effective	• Gaps in use of materials among AWWs (∼60%)
Supportive supervision		
Supervision visits in the past 6 mo	• Most FLWs (>90%) received supervision visits in the past 6 mo• More than half of FLWs reported use of supervision checklists• FLWs perceived supervision visits increased accountability and helped solve problems	• Less than half of FLWs were coached on MN or accompanied for home visits by supervisors• Vacancies in staff positions, particularly among ANM supervisors• Supervisors were not able to conduct regular visits due to high workload, meetings, and reporting duties
FLW knowledge		
Take IFA/calcium during pregnancy	• About 70–90% of FLWs knew about taking IFA/calcium for 6 mo during pregnancy	
Take IFA/calcium postpartum	• Most FLWs in I-ANC areas (>80%) knew about taking IFA/calcium for 6 mo during postpartum	
Benefit of IFA/calcium	• Around 60–70% knew about IFA benefits to reduce risk of anemia• Most FLWs (∼90%) in I-ANC areas knew about benefits of calcium for growth of children's bones	• Knowledge of other IFA/calcium benefits was low to moderate (30–50%)
Consume diverse diet	• ∼50–60% FLWs knew about consuming a diverse diet; ANMs in I-ANC areas had higher knowledge than those in S-ANC areas (62% compared with 36%)	• Moderate exposure to diet diversity knowledge
Knowledge of specific food groups	• Most FLWs knew about consuming food groups (>70%), including milk and green leafy vegetables	• Lower knowledge among ASHAs compared with AWWs and ANMs• Lower knowledge of animal source foods compared with other food groups
Recommended weight gain duringpregnancy	• Most FLWs (>70%) in I-ANC areas had correct knowledge of recommended weight gain during pregnancy	• Lower knowledge among AWWs and ASHAs (∼70%) compared with ANMs (90%)
Supply chain		
Distribution of IFA/calcium (frequency,quantity)	• FLWs distributed 50–60 IFA or calcium tablets to PW in the month preceding endline• Home delivery of IFA/calcium supplements by ASHAs	• Nearly 30% ANMs reported IFA and 50% ANMs reported calcium stock-out in last 3 mo preceding endline• Centralization of procurement in March 2019 from district to state caused delays• Inadequate storage facilities• Differing methods of demand estimation between chief pharmacist and medical officer at district• Program monitoring data showed inadequate IFA stock availability in 6 of 13 intervention blocks in the month preceding endline
Data use		
Monitor stock of IFA/calcium	• Most FLWs (>90%) collected data on PW	• Data use activities implemented toward end of project life
	• FLWs reported using monthly cluster meetings and HMIS data to monitor IFA stock and estimate demand• Supervision checklist data used to provide feedback on counseling	• Issues with HMIS server• Lack of staffing and technology access increased reporting burden for ICDS staff• Nearly 30% vacant positions among ANMs
	• Government data compared with program monitoring data to ensure data quality• Collaboration between health and ICDS departments to review data	• Data quality concerns—mistakes found in ANM reports• Review meetings not held regularly and mostly focused on administrative tasks, lack of time to discuss data
FLW service delivery		
Provision of IFA/calcium during VHSNDs	• Most FLWs (>90%) conducted VHSNDs	• Gaps in provision of IFA (50–60%) and calcium (40–60%) during VHSNDs
Provision of IFA/calcium during homevisits	• Most FLWs (>90%) conducted home visits	• Low provision of IFA (30–40%) and calcium (<30%) during home visits
Food demonstration during home visits	• All AWWs and ASHAs conducted home visits monthly (∼100%)	• Low provision of food demonstrations during home visits (<30%)
Weighing of PW at VHSNDs	• Most FLWs (>90%) conducted VHSNDs	• Gaps in weight-gain monitoring during VHSNDs, particularly among AWWs and ASHAs (∼50%)
FLW counseling—observed		
Take IFA/calcium during pregnancy	• Most FLWs (70–90%) counseled on taking IFA and more than half counseled on taking calcium during pregnancy	• Less than half of FLWs counseled on how to take calcium
How to take IFA/calcium	• Most FLWs (>70%) counseled on how to take IFA	
Benefit of IFA/calcium		• Only about a third of FLWs counseled on benefits of IFA/calcium
IFA/calcium side effects		• Only about a third of FLWs counseled on IFA side effectsLess than a fifth of FLWs counseled on calcium side effects
Consume diverse diet	• About 50–60% FLWs counseled on importance of diverse diet	• Lower counseling among ANMs compared with AWWs and ASHAs
Weight-gain monitoring		• Less than a third of FLWs counseled on weight-gain monitoring
Recommended weight gain		• Low to moderate counseling (40–60%) on recommended weight gain
FLW counseling—reported		
Take IFA/calcium during pregnancy	About 60–70% FLWs reported counseling on IFA/calcium	• Gaps in counseling on 180 IFA (40–50%) and 360 calcium (30–50%) supplements
Take IFA/calcium postpartum		• Only about a third of FLWs counseled on IFA/calcium during postpartum
How to take IFA/calcium		• Low to moderate counseling (30–50%) on how to consume IFA/calcium
Benefit of IFA/calcium	Around 50–70% counseled on benefits of calcium for growth of children's bones	• Less than half of FLWs counseled on other benefits of IFA/calcium
Consume diverse diet	∼70% of AWWs and ASHAs counseled about diverse diet	• Gaps in counseling among ANMs (∼45%)
Consume specific foods	Most FLWs (>80%) counseled on consuming green leafy vegetables daily	• Only about half of FLWs counseled on consuming animal source foods
Weight-gain monitoring		• Low to moderate counseling (30–50%) on weight-gain monitoring
Recommended weight gain		• Low to moderate counseling (40–60%) on recommended weight gain

1 ANM, auxiliary nurse midwife; ASHA, accredited social health activist; AWW, Anganwadi worker; FLW, frontline worker; HMIS, Health Management Information System; I-ANC, intensive antenatal care; ICDS, Integrated Child Development Services; IFA, iron and folic acid; MN, maternal nutrition; PW, pregnant women; S-ANC, standard antenatal care; VHSND, village health, sanitation, and nutrition day.

**TABLE 3:** Exposure to training among frontline workers, by intervention area and survey round^1^

	Baseline 2017		Endline 2019	
Characteristic	I-ANC area	S-ANC area	I-ANC area	S-ANC area
AWW	*n* = 91	*n* = 87	*n* = 87	*n* = 91
Received training in last year, %	—	—	95.4	94.5
Topics of training				
Counseling approach, %	24.1	26.5	48.3	37.4
How to engage husbands and family, %	17.2	8.8	16.1	8.8
Information on IFA/calcium, %	13.8	23.5	48.3	34.1
Information on diet, %	9.9	14.9	75.9	72.5
Information on weight gain, %	—	—	21.8	19.8
ASHA	*n* = 90	*n* = 90	*n* = 86	*n* = 88
Received training in last year, %	—	—	95.3	85.2*^2^
Topics of training				
Counseling approach, %	7.7	30	41.9	34.1
How to engage husbands and family, %	3.8	7.5	12.8	6.8
Any information on IFA/calcium, %	4.4	6.7	47.7	44.3
Information on diet, %	4.4	11.1	74.4	64.8
Any information on weight gain, %	—	—	31.4	23.9
ANM	*n* = 71	*n* = 68	*n* = 58	*n* = 69
Received training in last year, %	—	—	79.3	46.4***
Topics of training				
Counseling approach, %	21.4	21.6	34.5	17.4*
How to engage husbands and family, %	2.4	5.4	17.2	0.0**
Any information on IFA/calcium, %	11.9	18.9	51.7	21.7**
Information on diet, %	9.9	11.8	60.3	30.4**
Any information on weight gain, %	—	—	32.8	8.7**

1 Values are percentages. ANM, auxiliary nurse midwife; ASHA, accredited social health activist; AWW, Anganwadi worker; I-ANC, intensive antenatal care; IFA, iron and folic acid; S-ANC, standard antenatal care.

2 Asterisks indicate different from I-ANC area at that time: *P < 0.05, **P < 0.01, ***P < 0.001.

Qualitative interviews showed that FLWs in I-ANC areas had positive views of trainings and were able to better understand training content delivered through videos (Table 2).

They told everything very nicely. Actually, they told and showed things through video, so it was easy to understand. (ANM)

We were benefitted from the training. Earlier, we did not have much information about so many things but after training we came to know about Matritwa samiti [maternal committee] and maternal nutrition and our knowledge has increased a lot. (AWW)

Refresher trainings were not held regularly as planned during monthly meetings, however, as the focus was on administrative tasks. Nearly 30% of ANM positions were vacant, which limited coverage of training.

**FIGURE 2:**
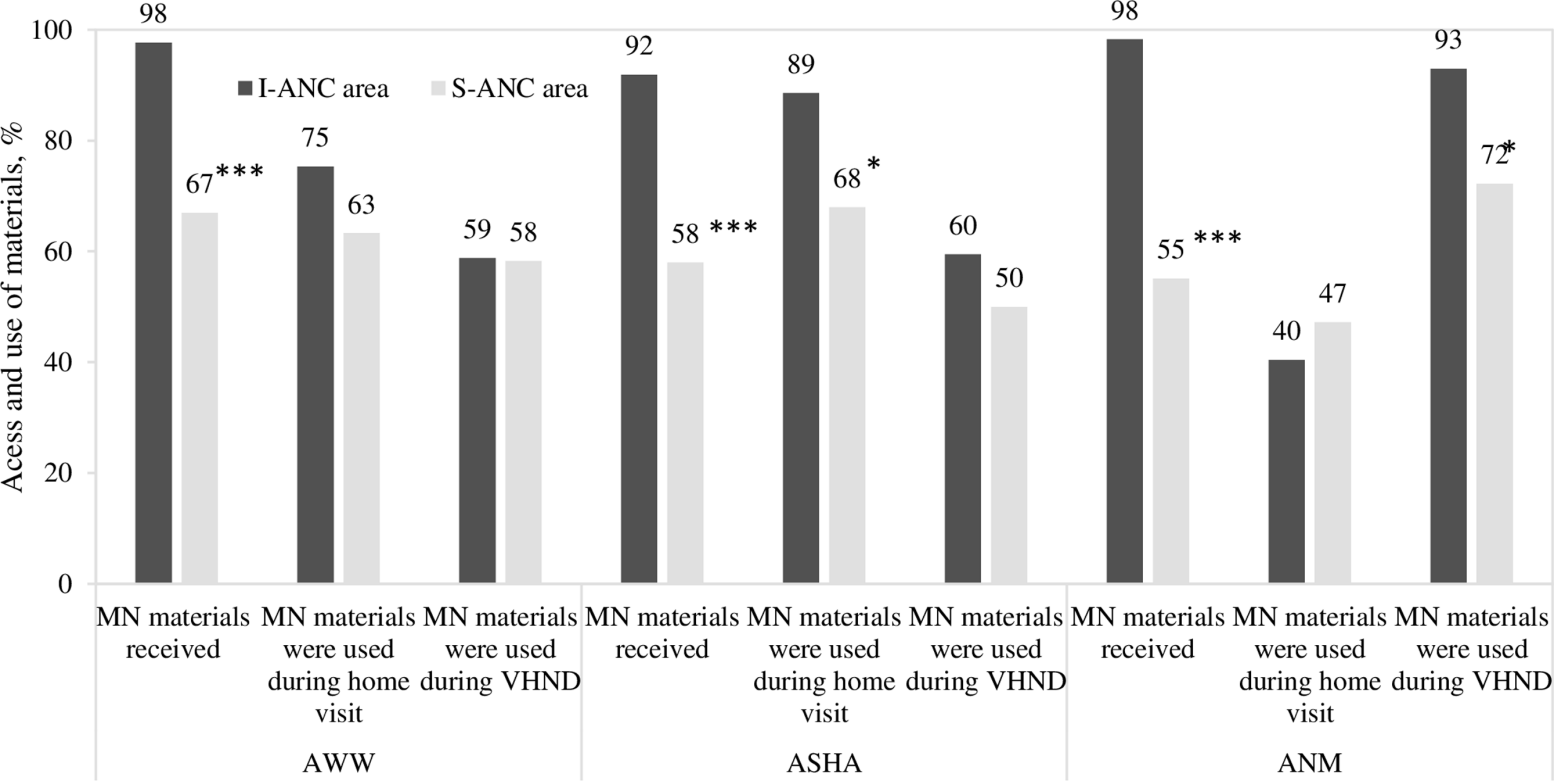
Access and use of materials among frontline workers, by intervention area at endline. Asterisks indicate different from I-ANC area: *P < 0.05, ***P < 0.001. ANM, auxiliary nurse midwife (*n* = 127); ASHA, accredited social health activist (*n* = 174); AWW, Anganwadi worker (*n* = 178); I-ANC, intensive antenatal care; MN, maternal nutrition; S-ANC, standard antenatal care; VHSND, village health, sanitation, and nutrition day.

Overall, access to maternal nutrition materials was high in both areas, but use of materials was limited during contacts with pregnant women. FLWs in I-ANC areas were given a maternal nutrition calendar and flipcharts (98% for AWWs and ANMs and 92% for ASHAs) to aid them with counseling. FLWs in S-ANC areas also had access to job aids but at lower coverage (67% for AWWs, 58% for ASHAs, and 55% for ANMs) (**Figure 2**). More ASHAs in I-ANC areas used job aids during home visits (89% compared with 68%), and more ANMs used job aids on VHSNDs (93% compared with 72%) than their counterparts in S-ANC areas. Use of materials was lower among AWWs and similar between areas.

FLWs in I-ANC areas had positive views of maternal nutrition materials and job aids, which they reported helped to provide more effective counseling.

Initially we faced problem while making them understand but gradually they understand. We have provided them calendar and those calendars are hanging in their homes. So, if they wouldn't be able to understand orally then we explain to them through pictures of the calendar. (AWW)

### Strengthening supportive supervision for FLWs

**FIGURE 3:**
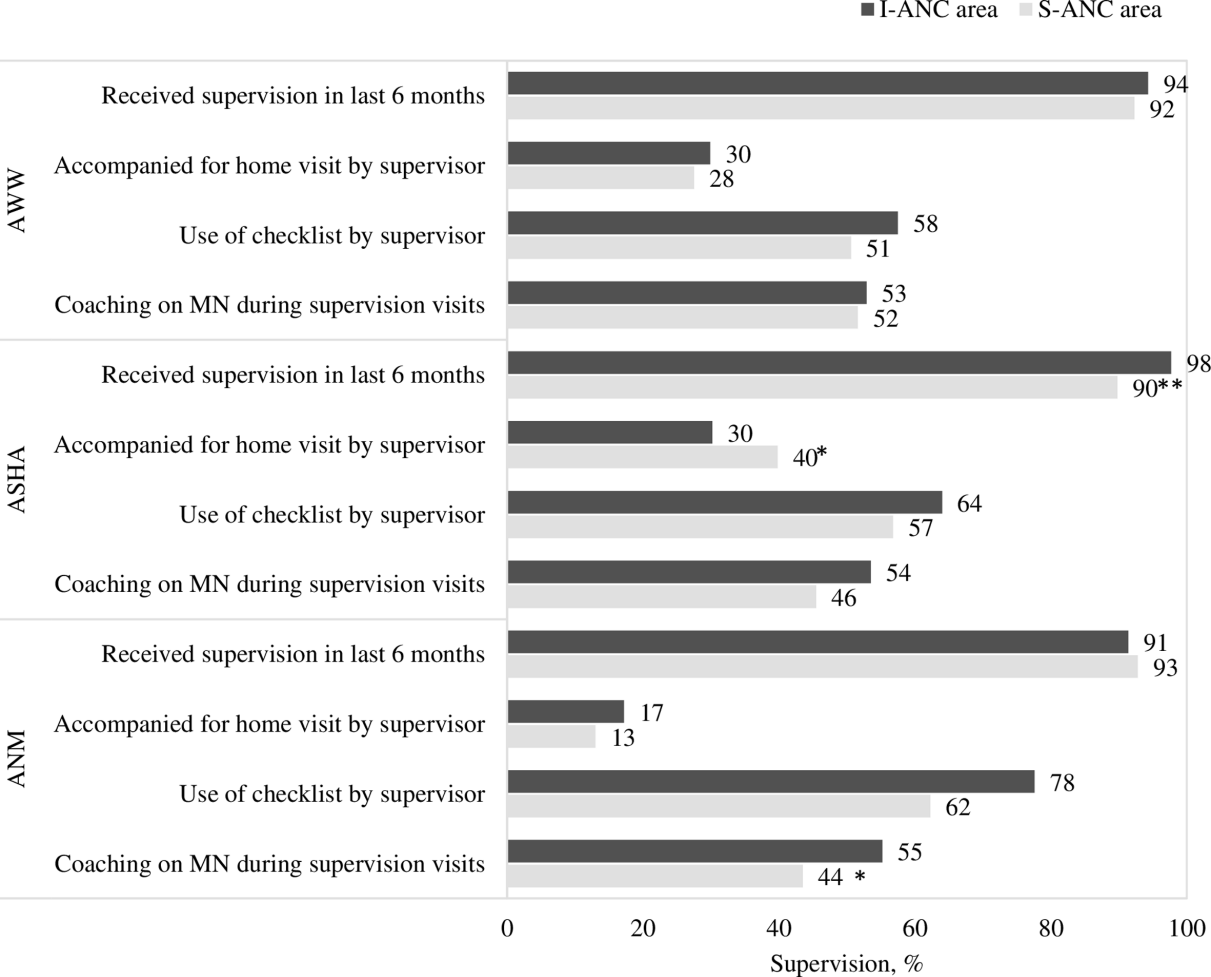
Supervision among frontline workers, by intervention area at endline. Asterisks indicate different from I-ANC area: *P < 0.05, **P < 0.01. ANM, auxiliary nurse midwife (*n* = 127); ASHA, accredited social health activist (*n* = 174); AWW, Anganwadi worker (*n* = 178); MN, maternal nutrition; I-ANC, intensive antenatal care; S-ANC, standard antenatal care.

Overall, receipt of supervision visits was high in I-ANC and S-ANC areas, but gaps were prevalent in key supportive supervision practices. Most FLWs (>90%) in both areas received supervisory visits, but only around half of FLWs reported being coached on maternal nutrition during visits and a third of AWWs and ASHAs being accompanied for home visits by their supervisors (**Figure 3**). ANMs in I-ANC areas were more likely to receive supervision through use of a checklist compared with those in S-ANC areas (78% compared with 62%). Qualitative interviews showed that FLWs in I-ANC areas valued supervision visits in helping them solve problems and increasing their accountability. Supportive supervision was limited, however, by vacancies among ICDS supervisory staff and supervisors being unable to conduct regular visits due to competing work priorities.

It is good when the supervisor come for the visit, this is how we get a lot of information and can also clear our doubts, like when a supervisor comes for the visit twice a month then she check our work this is how we are also scared and do the work timely and also clear the doubts. (ASHA)

### FLW knowledge

Knowledge of consuming iron–folic acid and calcium supplements during pregnancy was similarly high in both areas at endline (70–80%), but gaps were prevalent on knowledge of supplement benefits. Knowledge of consuming iron–folic acid supplements during postpartum improved more among AWWs and ASHAs in I-ANC than in S-ANC areas [DID: 35 and 39 percentage points (pp), both *P* < 0.001] (**Table 4**). Most FLWs knew about iron–folic acid benefits of reducing the risk of anemia (∼60–70%), but knowledge of other benefits was low to moderate (30–50%) in both areas. ASHAs in I-ANC areas had higher knowledge of iron–folic acid benefits to reduce risk of low birth weight than those in S-ANC areas (DID: 33 pp, *P* < 0.01).

**TABLE 4:** Frontline workers knowledge, by intervention area and survey round^1^

	Baseline 2017		Endline 2019		
Characteristic	I-ANC area	S-ANC area	I-ANC area	S-ANC area	DID
AWW	*n* = 91	*n* = 87	*n* = 87	*n* = 91	
IFA					
Take IFA for 6 mo during pregnancy	46.2	62.1*^2^	83.9	81.3	18.6^+^^3^
Take IFA for 6 mo during postpartum	20.9	25.3	87.4	57.1***	34.8^###^
Reasons why PW should take IFA					
To reduce risk of anemia for PW	70.3	70.1	72.4	73.6	–1.7
To reduce risk of anemia for the child	59.3	51.7	65.5	61.5	–3.7
To reduce risk of low birth weight	19.8	20.7	48.3	38.5	10.3
To help improve child's intelligence	20.9	25.3	40.2	26.4*	18.1^+^
To reduce risk of excessive blood loss during/after delivery	42.9	34.5	54.0	54.9	–9.0
Calcium					
Take calcium for 6 mo during pregnancy	18.7	16.1	79.3	65.9**	10.9
Take calcium for 6 mo during postpartum	8.8	10.3	85.1	51.7***	35.0^###^
Reasons why PW should take calcium					
To recover the loss in pregnant woman's body	18.7	21.8	46.0	37.4	12.1
To ensure growth of child's bones and teeth	37.4	40.2*	92.0	64.8***	29.7^##^
To reduce the risk of high blood pressure, eclampsia	20.9	18.4	60.9	36.3**	22.0^#^
Dietary diversity					
Eat 5 varieties of foods in addition to rice and dal	28.6	44.8	56.3	50.5	22.1
Eat fish/meat daily	40.7	41.4	71.3	50.5**	21.5^#^
Eat egg daily, if acceptable	48.4	47.1	66.7	47.3*	18.0
Take milk/milk product daily	64.8	65.5	74.7	80.2	–5.0
Eat green leafy vegetables daily	83.5	75.9	79.3	81.3	–9.7
Eat yellow/orange vegetables/fruit daily	63.7	54.0	60.9	67.0	–16.2
Weight gain					
10–12 kg during pregnancy	39.6	32.2	72.4	42.9***	22.6^#^
ASHA	*n* = 90	*n* = 90	*n* = 86	*n* = 88	
IFA					
Take IFA for 6 mo during pregnancy	57.8	54.4	84.9	88.6	–7.4
Take IFA for 6 mo during postpartum	18.9	26.7	88.4	56.8***	39.4^###^
Reasons why PW should take IFA					
To reduce risk of anemia for PW	60.0	65.6	68.6	77.3	–2.5
To reduce risk of anemia for the child	57.8	64.4	64.0	64.8	5.8
To reduce risk of low birth weight	16.7	21.1	61.6	33.0***	33.0^##^
To help improve child's intelligence	15.6	23.3	33.7	30.7	10.1
To reduce risk of excessive blood loss during/after delivery	38.9	42.2	52.3	51.1	3.9
Calcium					
Take calcium for 6 mo during pregnancy	14.4	16.7	83.7	69.3**	16.6
Take calcium for 6 mo during postpartum	0.0	0.0	79.1	48.9***	30.2^##^
Reasons why PW should take calcium					
To recover the loss in pregnant woman's body	22.2	22.2	47.7	43.2	4.4
To ensure growth of child's bones and teeth	25.6	36.7	88.4	71.6**	28.0^#^
To reduce the risk of high blood pressure, eclampsia	14.4	23.3*	61.6	35.2***	36.1^###^
Dietary diversity					
Eat 5 varieties of foods in addition to rice and dal	33.3	27.8	58.1	46.6	5.9
Eat fish/meat daily	30.0	38.9	57	59.1	7.1
Eat egg daily, if acceptable	37.8	45.6	59.3	51.1	15.1
Take milk/milk product daily	68.9	68.9	67.4	85.2	–18.9
Eat green leafy vegetables daily	74.4	76.7	67.4	83.0	–13.5
Eat yellow/orange vegetables/fruit daily	60.0	50.0	53.5	72.7	–29.4
Weight gain					
10–12 kg during pregnancy	30.0	35.6	74.4	56.8*	22.9^#^
ANM	*n* = 71	*n* = 68	*n* = 58	*n* = 69	
IFA					
Take IFA for 6 mo during pregnancy	63.4	60.3	91.4	94.2	–5.6
Take IFA for 6 mo during postpartum	38.0	33.8	91.4	69.6**	17.1
Reasons why PW should take IFA					
To reduce risk of anemia for PW	71.8	72.1	58.6	71.0	–11.9
To reduce risk of anemia for the child	63.4	67.6	81.0	73.9	11.2
To reduce risk of low birth weight	33.8	29.4	48.3	36.2	6.7
To help improve child's intelligence	31.0	38.2	37.9	34.8	11.4
To reduce risk of excessive blood loss during/after delivery	40.8	47.1	56.9	42.0*	21.7
Calcium					
Take calcium for 6 mo during pregnancy	32.4	35.3	89.7	88.4**	5.1
Take calcium for 6 mo during postpartum	19.7	25.0	81.0	71.0	15.2
Reasons why PW should take calcium					
To recover the loss in pregnant woman's body	32.4	47.1	41.4	47.8	9.3
To ensure growth of child's bones and teeth	70.4	80.9*	94.8	94.2	11.5
To reduce the risk of high blood pressure, eclampsia	28.2	35.3	31.0	27.5	10.6
Dietary diversity					
Eat 5 varieties of foods in addition to rice and dal	31.0	41.2	62.1	36.2**	36.0^#^
Eat fish/meat daily	47.9	42.6	51.7	42.0	6.3
Eat egg daily, if acceptable	56.3	51.5	69.0	36.2***	26.7^#^
Take milk/milk product daily	70.4	66.2	70.7	71.0	–6.8
Eat green leafy vegetables daily	76.1	76.5	84.5	87.0	–1.1
Eat yellow/orange vegetables/fruit daily	54.9	58.8	77.6	66.7	15.6
Weight gain					
10–12 kg during pregnancy	52.1	55.9	91.4	68.1**	25.6^#^

1 Values are percentages. ANM, auxiliary nurse midwife; ASHA, accredited social health activist; AWW, Anganwadi worker; DID, difference-in-differences; I-ANC, intensive antenatal care; IFA, iron and folic acid; PW, pregnant women; S-ANC, standard antenatal care.

2 Asterisks indicate different from I-ANC area at that time: *P < 0.05, **P < 0.01, ***P < 0.001.

3 DID effect estimates between baseline and endline: ^+^*P* < 0.1, ^#^  *P* < 0.05, ^##^  *P* < 0.01, ^###^  *P* < 0.001.

Improvement in knowledge of calcium supplements during postpartum was higher in I-ANC compared with S-ANC areas for AWWs and ASHAs (DID: 35 and 30 pp, *P* < 0.001 and *P* < 0.01, respectively) and was similar for ANMs in both areas (∼75%). Although most FLWs knew about calcium benefits for growth of children's bones, knowledge of other benefits was low to moderate (30–50%). AWWs and ASHAs in I-ANC compared with S-ANC areas had higher knowledge of the benefits of calcium to ensure growth of children's bones (DID: ∼29 pp, *P* < 0.01 and *P* < 0.05, respectively) and to reduce the risk of high blood pressure/fits (DID: 22 and 36 pp, *P* < 0.05 and *P* < 0.001, respectively).

FLWs’ knowledge of diet diversity was moderate in I-ANC and S-ANC areas, with ∼50–60% knowing about consuming 5 or more food groups during pregnancy; knowledge of animal source foods was lower in both areas. Knowledge of diet diversity was similar for AWWs and ASHAs in both areas and higher for ANMs in I-ANC compared with S-ANC areas (DID: 36 pp, *P* < 0.05). For specific foods, most FLWs (>70%) in both areas knew about consuming milk and green leafy vegetables, but knowledge of animal source foods was lower. AWWs and ANMs in I-ANC areas had higher knowledge than those in S-ANC areas about consuming eggs and fish/meat daily during pregnancy (DID: 22 pp and 27 pp, respectively, both *P* < 0.05).

Knowledge of weight gain improved over time in both areas, with higher improvements among all FLWs in I-ANC areas compared with those in S-ANC areas (DID: 23–25 pp, all *P* < 0.05). Most FLWs (>70%) in I-ANC areas had correct knowledge of recommended weight gain during pregnancy (10–12 kg), but this knowledge was lower among AWWs and ASHAs (∼70%) compared with ANMs (90%).

### Strengthening of iron–folic acid and calcium supplementation supply chain

Major supply chain bottlenecks related to iron–folic acid and calcium supplements were observed in both I-ANC and S-ANC areas. Interventions related to supply chain were not restricted in I-ANC areas only but implemented at the state or district level and specifically related to forecasting and procurement of supplements. Similar changes were observed over time for FLWs in both areas for supplement provision. FLWs reported providing 50–60 iron–folic acid and calcium tablets a month to pregnant women, but nearly 30% and 50% of ANMs reported stock-outs of iron–folic acid and calcium supplements in the past 3 months, respectively. A&T program monitoring data showed inadequate stock in half of I-ANC blocks for iron–folic acid and in all I-ANC blocks for calcium supplements in the month preceding endline (Table 2).

ASHAs described how they delivered supplements at home to pregnant women who did not attend ANC during VHSNDs, which was highlighted as a positive step in improving provision of iron–folic acid and calcium. Qualitative findings also emphasized logistic issues along the supply chain, including delays due to centralization of procurement, differing methods of demand estimation between government staff, and inadequate storage facilities.

Both of the districts had a decent amount of iron–folic acid until the end of 2018, but the shift in procurement [from district to state] did start delaying iron–folic acid procurement. (A&T program staff)

For most of the medicines, not just for iron–folic acid and calcium, the quarterly purchase cycle was because the storehouse in Unnao was smaller and could not accommodate a 6-month or annual cycle. (A&T program staff)

From October onwards, we started to see VHSNDs without calcium . . . in Unnao the distribution was pretty haphazard . . . most of the blocks were out of calcium by January. (A&T program staff)

### Strategic use of data to track the progress of interventions and identify needed improvements

Data use was higher in I-ANC compared with S-ANC areas, but key gaps were prevalent in the strategic use of data during monthly review meetings in both areas. Use of data was higher in I-ANC than S-ANC areas during monthly meetings (66% compared with 50% among AWWs and 58% compared with 42% among supervisors), during cluster review meetings (52% compared with 36% among AWWs), and for decision-making purposes (23% compared with 15% among supervisors) (**Table 5**). Block-level staff described using Health Management Information Systems data to monitor iron–folic acid and calcium stocks and estimate demand. Supervisors in I-ANC areas reported using supportive supervision checklist data to provide feedback to FLWs on maternal nutrition counseling, including gaps and areas of improvement. Collaboration between government staff from different departments to review data was also highlighted as a positive step.

**TABLE 5:** Data use among frontline workers and supervisors, by intervention area at endline^1^

Characteristic	I-ANC area, %	S-ANC area, %
AWW	*n* = 87	*n* = 91
Data discussed in AAA meetings	65.5	49.5*^2^
Data discussed in sector/cluster review meeting	51.7	36.3*
Data used to monitor stock of IFA and/or calcium supplements	39.1	37.4
Data used to identify areas for improvement and gaps	35.6	34.1
Data used for decision making on areas for improvement	28.7	25.3
Challenges in using data		
Data are difficult to understand	23	19.8
Do not feel data are accurate/problems in data quality	16.1	20.9
Do not feel use of data is important	12.6	7.7
Lack of time for interpreting/discussing data	13.8	13.2
ASHA	*n* = 86	*n* = 88
Data discussed in AAA meetings	58.1	56.8
Data discussed in sector/cluster review meeting	40.7	34.1
Data used to monitor stock of IFA and/or calcium supplements	48.8	47.7
Data used to identify areas for improvement and gaps	33.7	37.5
Data used for decision making on areas for improvement	29.1	27.3
Challenges in using data		
Data are difficult to understand	26.7	23.9
Do not feel data are accurate/problems in data quality	14	15.9
Do not feel use of data is important	14	10.2
Lack of time for interpreting/discussing data	11.6	17
ANM	*n* = 58	*n* = 69
Data discussed in AAA meetings	58.6	71.0
Data discussed in sector/cluster review meeting	65.5	49.3
Data used to monitor stock of IFA and/or calcium supplements	48.3	37.7
Data used to identify areas for improvement and gaps	60.3	50.7
Data used for decision making on areas for improvement	27.6	26.1
Challenges in using data		
Data are difficult to understand	13.8	1.4**
Do not feel data are accurate/problems in data quality	8.6	4.3
Do not feel use of data is important	3.4	0.0
Lack of time for interpreting/discussing data	13.8	5.8
Supervisors	*n* = 103	*n* = 106
Data discussed in AAA meetings	58.3	41.5*
Data discussed in sector/cluster review meeting	61.2	50.9
Data used to monitor stock of IFA and/or calcium supplements	32	32.1
Data used to identify areas for improvement and gaps	49.5	44.3
Data used for decision making on areas for improvement	23.3	15.1*
Challenges in using data		
Data are difficult to understand	15.5	10.4
Do not feel data are accurate/problems in data quality	14.6	6.6**
Do not feel use of data is important	4.9	4.7
Lack of time for interpreting/discussing data	9.7	5.7

1 Values are percentages. AAA, auxiliary nurse midwife, accredited social health activist, and Anganwadi worker; ANM, auxiliary nurse midwife; ASHA, accredited social health activist; AWW, Anganwadi worker; I-ANC, intensive antenatal care; IFA, iron and folic acid; MN, maternal nutrition; S-ANC, standard antenatal care.

2 Asterisks indicate different from I-ANC area: *P < 0.05, **P < 0.01.

We will sometimes look at the trends in ANC registration in the first trimester from the past 2–3 years to see how many pregnant women are in the village. That way we can estimate how much iron–folic acid and calcium we need in the block. (Block-level staff)

Through the checklist, I see what ASHA has told [the pregnant woman] . . . we ask ASHA if she has done [counseling]. If she says yes, we write yes and if she has not done, we write no . . . [if she writes no] I explain to her why she has not talked about the topic and then give her the information. (Supervisor)

Challenges in using data were reported by ∼10–25% FLWs and 5–15% supervisors in both areas, with key challenges including difficulty in understanding data, poor data quality, and lack of time. Qualitative data highlighted additional constraints in data use, including server issues, staff vacancies, lack of technology, and reporting burden. In addition, monthly review meetings mostly focused on administrative tasks, which limited time to discuss data.

### FLW service delivery

Most service provision improved in both areas over time, but critical gaps remained in I-ANC and S-ANC areas on fully using service delivery platforms to provide iron–folic acid and calcium supplements, conduct food demonstration, and weigh pregnant women. Higher support to ANMs and mobilization by ASHAs for ANC checkups were observed in I-ANC compared with S-ANC areas (34 pp) (**Table 6**). During monthly VHSNDs, ∼60% of AWWs and ASHAs and 75% of ANMs provided iron–folic acid supplements for pregnant women at endline, but calcium provision was much lower. All ASHAs and ANMs conducted monthly home visits to pregnant women, but less than a third provided iron–folic acid and calcium supplements during home visits.

**TABLE 6:** Service delivery by frontline workers, by intervention area and survey round^1^

	Baseline 2017		Endline 2019		
Characteristic	I-ANC area	S-ANC area	I-ANC area	S-ANC area	DID
AWW	*n* = 91	*n* = 87	*n* = 87	*n* = 91	
VHSND conducted at least once a month	92.3	96.6	93.1	95.6	1.8
VHSND services provided					
Provision of IFA	45.1	47.1	56.3	64.8	–6.7
Provision of calcium	3.3	8.1	48.3	38.5	14.7
Weighing of PW	60.4	58.6	58.6	65.9	–9.3
Home visit to PW at least once a month	51.7	54.0	98.9	100	1.5
Services provided during home visits to PW					
Provide free IFA	22.0	36.8	19.5	25.3	8.8
Provide free calcium	4.4	11.5	24.1	14.3	16.7
Counsel PW about taking IFA	58.2	56.3	47.1	35.2	9.9
Counsel PW about taking calcium	8.8	17.2	32.2	22.0	18.9
Advice on maternal nutrition	54.9	59.8	62.1	65.9	1.1
Food demonstration	23.1	20.7	27.6	28.6	–3.3
Provide weight-gain advice during pregnancy	27.5	29.9	33.3	28.6	7.1
ASHA	*n* = 90	*n* = 90	*n* = 86	*n* = 88	
VHSND conducted at least once a month	88.9	90.0	96.5	94.3	3.1
VHSND services provided					
ANC checkup	27.7	41.7	55.8	35.2**^2^	33.5^##^^3^
Provision of IFA	44.4	44.4	57.0	55.7	1.6
Provision of calcium	8.9	6.7	45.4	42.1	1.1
Weighing of PW	54.4	55.6	55.8	55.7	1.4
Home visit to PW at least once a month	55.6	54.4	100	100	–1.1
Services provided during home visits to PW					
Provide free IFA	38.9	40.0	37.2	33.0	6.0
Provide free calcium	6.7	5.6	23.3	23.9	–1.7
Counsel PW about taking IFA	53.3	52.2	54.7	47.7	4.9
Counsel PW about taking calcium	17.8	10.0	39.5	33.0	–1.3
Advice on maternal nutrition	53.3	53.3	52.3	67.1	–14.5
Food demonstration	12.2	24.4	26.7	29.5	9.5
Provide weight-gain advice during pregnancy	13.3	25.6	36.0	34.1	14.6
ANM	*n* = 71	*n* = 68	*n* = 58	*n* = 69	
VHSND conducted at least once a month	94.4	98.5	98.3	98.6	3.7
VHSND services provided					
ANC checkup	58.8	56.7	48.3	53.6	–5.7
Provision of IFA	47.9	45.6	75.9	85.5	–11.6
Provision of calcium	2.8	5.9	65.5	66.7	1.9
Weighing of PW	46.5	60.3	72.4	79.7	5.2

1 Values are percentages. ANC, antenatal care; ANM, auxiliary nurse midwife; ASHA, accredited social health activist; AWW, Anganwadi worker; DID, difference-in-differences; I-ANC, intensive antenatal care; IFA, iron and folic acid; PW, pregnant women; S-ANC, standard antenatal care; VHSND, village health, sanitation, and nutrition day.

2 Asterisks indicate different from I-ANC area at that time: **P < 0.01.

3 DID effect estimates between baseline and endline: ^##^  *P* < 0.01.

Although ∼60% of AWWs and ASHAs provided nutrition counseling during home visits, less than a third conducted food demonstrations to help women understand which foods they should consume during pregnancy. These findings were similar between I-ANC and S-ANC areas.

Less than 60% of AWWs and ASHAs monitored weight during VHSNDs, similar in both areas. During home visits, only about a third of AWWs and ASHAs in both areas provided weight-gain advice during pregnancy. Higher percentage of ANMs weighed women during VHSNDs (∼75%), and no difference was found between study areas.

### FLW counseling

Despite improvements over time, critical gaps were prevalent for specific counseling on supplement benefits (∼30%), diet diversity (50–60%), and weight-gain monitoring (30–40%) in I-ANC and S-ANC areas. Findings from counseling observations showed that most FLWs advised women to take iron–folic acid regularly during pregnancy (60–75% among AWWs and ASHAs and >80% among ANMs) (**Table 7**), but the percentage of FLWs who advised women to take calcium was lower (33–46% among AWWs and ASHAs and 53–63% among ANMs). Several gaps remained in both areas in counseling on how to consume iron–folic acid (40–57% among AWWs and ASHAs), benefits of iron–folic acid (26–46%), and side effects and how to manage them (12–43%). Discussions on benefits and side effects of calcium were even lower and similar among FLWs in both areas. AWWs and ASHAs in I-ANC areas were more likely to advise women on how to take calcium compared with those in S-ANC areas (47% compared with 26%). Reported counseling on iron–folic acid and calcium showed similar patterns as observations, with some higher counseling in I-ANC compared with S-ANC areas for consuming supplements during pregnancy, how to consume, and benefits of consumption (**Supplemental Tables 2–3**).

**TABLE 7:** Observed counseling, by intervention area at endline^1^

Characteristic	I-ANC area, %	S-ANC area, %
AWW	*n* = 81	*n* = 85
IFA		
Advised PW to take IFA regularly (1 tablet/d)	74.1	61.2
Advised to take IFA at nighttime with water or lemon water	45.7	40.0
Explained/reminded about any IFA benefits	29.6	25.9
Discussed side effects that may occur and how to manage them	23.5	11.8
Addressed any IFA supply gap by providing supplies/referring to ANM	14.8	17.6
Reminded woman not to take IFA with tea or coffee or milk	29.6	30.6
Calcium		
Advised PW to take calcium regularly (2 tablets/d)	44.4	32.9
Advised PW to take calcium in the morning and afternoon after food	46.9	25.9**^2^
Explained/reminded about any of calcium benefits	18.5	11.8
Discussed how to manage any calcium-related side effects	8.6	7.1
Addressed any calcium supply gap by providing supplies/referring to ANM	9.9	9.4
Diet diversity		
Asked women about foods consumed in past 24 h and helped to add missing fooditems using locally available nutritious foods	62.4	54.1
Counseled on importance of diverse diet	61.7	60.0
Advised women on consuming at least 5 recommended food groups in a day	81.5	87.1
Weight-gain monitoring		
Checked whether weight gain is adequate using measurements in MCP card	13.6	18.8
Explained that a woman should gain 10–12 kg weight during pregnancy	28.4	24.7
Counseled on the importance of weight gain during pregnancy	37.0	45.9
ASHA	*n* = 81	*n* = 85
IFA		
Advised PW to take IFA regularly (1 tablet/d)	76.5	74.1
Advised to take IFA at nighttime with water or lemon water	56.8	50.6
Explained/reminded about any IFA benefits	34.6	34.1
Discussed side effects that may occur and how to manage them	22.2	18.8
Addressed any IFA supply gap by providing supplies/referring to ANM	17.3	17.6
Reminded woman not to take IFA with tea or coffee or milk	28.4	40.0
Calcium		
Advised PW to take calcium regularly (2 tablets/d)	45.7	35.3
Advised PW to take calcium in the morning and afternoon after food	46.9	27.1*
Explained/reminded about any of calcium benefits	22.2	11.8
Discussed how to manage any calcium-related side effects	14.8	5.9
Addressed any calcium supply gap by providing supplies/referring to ANM	13.6	14.1
Diet diversity		
Asked women about foods consumed in past 24 h and helped to add missing food items using locally available nutritious foods	54.3	55.3
Counseled on importance of diverse diet	61.7	60.0
Advised women on consuming at least 5 recommended food groups in a day	80.3	89.4
Weight-gain monitoring		
Checked whether weight gain is adequate using measurements in MCP card	25.9	27.1
Explained that a woman should gain 10–12 kg weight during pregnancy	29.6	34.1
Counseled on the importance of weight gain during pregnancy	48.1	55.3
ANM	*n* = 35	*n* = 40
IFA		
Advised PW to take IFA regularly (1 tablet/d)	80.0	95.0
Advised to take IFA at nighttime with water or lemon water	68.6	67.5
Explained/reminded about any IFA benefits	45.7	30.0
Discussed side effects that may occur and how to manage them	42.9	25.0
Reminded woman not to take IFA with tea or coffee or milk	42.9	25.0
Calcium		
Advised PW to take calcium regularly (2 tablets/d)	62.9	52.5
Advised PW to take calcium in the morning and afternoon after food	60.0	37.5
Explained/reminded about any of calcium benefits	37.1	20.0
Discussed how to manage any calcium-related side effects	31.4	15.0
Diet diversity		
Counseled on importance of diverse diet	74.1	71.0
Advised women on consuming at least 5 recommended food groups in a day	87.9	87.0
Weight-gain monitoring		
Checked whether weight gain is adequate using measurements in MCP card	28.6	20.0
Explained that a woman should gain 10–12 kg weight during pregnancy	31.4	25.0
Counseled on the importance of weight gain during pregnancy	71.4	50.0

1 Values are percentages. ANM, auxiliary nurse midwife; ASHA, accredited social health activist; AWW, Anganwadi worker; I-ANC, intensive antenatal care; IFA, iron and folic acid; MCP, mother and child protection; PW, pregnant women; S-ANC, standard antenatal care.

2 Asterisks indicate different from I-ANC area: *P < 0.05, **P < 0.01.

Counseling observations for diet diversity showed ∼50–60% of FLWs counseled on the importance of a diverse diet, and 70–80% counseled on consuming at least 5 food groups in a day, similar between I-ANC and S-ANC areas (Table 7). In addition, ∼60% of AWWs and ASHAs in both areas enquired about foods pregnant women consumed in the previous 24 h during counseling sessions. Reported counseling on diet diversity showed similar patterns as observations, with some improvements in I-ANC compared with S-ANC areas for counseling on animal source foods during pregnancy (**Supplemental Table 4**).

Counseling observations for weight monitoring showed gaps at endline and were nondifferential between study areas (Table 7). Less than a quarter of FLWs checked whether weight gain was adequate using measurements recorded in the Mother and Child Protection card; only around a third of FLWs explained optimal gestational weight gain to women. Counseling on the importance of weight gain during pregnancy was 37–55% among AWWs and ASHAs and 50–71% among ANMs. Reported counseling on weight-gain monitoring showed patterns similar to the observations, with some improvements in I-ANC compared with S-ANC areas for counseling on weight-gain monitoring during VHSNDs and recommended weight gain (**Supplemental Table 5**).

## Discussion

To address the challenges of maternal undernutrition, A&T implemented a bundled package of maternal nutrition interventions, but the impact evaluation only found limited impacts on key outcomes related to consumption of micronutrient supplements, diet diversity, and gestational weight gain. Using mixed methods and a theory-driven PIP analysis, this study identified successes and challenges along PIP domains for a bundled package of maternal nutrition: micronutrient supplementation, diet diversity counseling, and weight-gain monitoring. We observed secular trends in several domains along the PIP in both intervention areas and found greater improvements in some domains in I-ANC compared with S-ANC areas. Across interventions, common areas of successes included high coverage of training, receipt and use of maternal nutrition job aids among FLWs in I-ANC areas, and high coverage of VHSNDs and home visits. Critical challenges for all interventions included staff vacancies among ANMs and ICDS supervisors, inadequate refresher trainings, gaps in supportive supervision practices, and limited use of data due to technical issues and lack of time. The PIP analysis highlighted critical gaps in FLW knowledge, service delivery, counseling, and supply chain challenges that help explain the limited impacts seen in the study outcomes.

Across the 3 types of FLWs, ANMs in I-ANC compared with S-ANC areas showed the greatest improvements in exposure to maternal nutrition training content. ASHAs and ANMs in I-ANC compared with S-ANC areas were more likely to use maternal nutrition materials, whereas use of materials was similar among AWWs in both areas. Knowledge of and counseling on iron–folic acid and calcium supplements were low to moderate among AWWs and moderate to high among ASHAs and ANMs, with some improvements in I-ANC compared with S-ANC areas. For diet diversity, knowledge and counseling were moderate for AWWs, ASHAs, and ANMs with some higher knowledge in I-ANC compared with S-ANC areas among AWWs and ANMs. Knowledge of and counseling on weight gain were moderate and higher in I-ANC compared with S-ANC areas for all FLWs.

We also identified key successes and challenges for each specific intervention. For micronutrient supplementation, successes included FLW knowledge of and counseling on taking iron–folic acid and calcium during pregnancy. Key challenges were limited exposure to training content on iron–folic acid and calcium, low knowledge of supplement benefits, supply chain stock-outs, gaps in provision of iron–folic acid and calcium, and counseling on supplement benefits and side effects. Intervention-specific successes for diet diversity counseling included training content on diet counseling, FLW knowledge of diet diversity and importance of dark green leafy vegetables, and counseling on the importance of diet. Challenges for diet diversity counseling included low knowledge of animal source foods, food demonstrations during home visits, and low counseling on specific food groups. For weight-gain monitoring, most FLWs had correct knowledge of recommended weight gain during pregnancy, but critical challenges included training on weight-gain monitoring, weighing of women during VHSNDs, and counseling on recommended weight gain and weight monitoring.

Across maternal nutrition interventions, exposure to training was high overall, but gaps occurred in training content. Monthly meetings were available as a platform for refresher training but not fully used, with irregular meetings and overemphasis on administrative tasks, reducing time for technical content discussion. Previous literature has highlighted the importance of regular continuous trainings to improve FLW knowledge and skills and deliver quality services (11, 29); strengthening monthly meetings as an active platform for refresher trainings to exchange experiences and fill in knowledge gaps is critical. Ensuring that training manuals are comprehensive and include context-appropriate information for problem solving is important for improving content (19). Although FLWs had positive perceptions of job aids, only around two-thirds of them used materials. Ensuring that materials are concise and easily understandable by FLWs is important so that use of materials is not obstructed due to lack of time (20). Improving knowledge of FLWs to deliver services is a critical next step following training. Overall knowledge was moderate to high but with gaps in detailed knowledge of supplement benefits and recommended foods during pregnancy. Tailored frequent trainings are important to equip FLWs with the knowledge to provide accurate counseling to mothers.

Although supportive supervision was found to be a key intervention element influencing FLWs’ performance in LMICs (29), high reporting burden and focus on administrative tasks among supervisors and block-level staff obstructed supportive supervision practices and effective use of data in our study. Potential improvements include providing constructive feedback, coaching, and problem solving during visits instead of only monitoring and finding faults (20). A change in system culture is needed to shift the focus from administrative functions toward training and coaching support for improvements in FLW performance. Literature on strategic use of data to track intervention progress and identify areas of improvement is limited, and this topic is important to study in future research, as well-resourced nutrition data and information systems are essential for effective implementation (15, 27). Setting up monthly or quarterly review systems at the district and block levels can be used to track performance, supplement stock, and identify bottlenecks. The study findings showed the potential of monthly meeting platforms but also highlighted challenges of regularity in their functioning.

Availability of supplements and a well-functioning supply chain are essential for micronutrient supplementation interventions. A study from Kenya examined the integration of iron–folic acid and calcium supplements into primary health care and found substantial supply chain bottlenecks, particularly for calcium supplements (30). Delays were prevalent in demand estimation of supplements, which caused stock-outs in some areas (30). Our study reported similar issues with the supply chain for both iron–folic acid and calcium supplements, especially the latter. One such bottleneck in the supply chain identified through our study was differing methods of stock estimation at different levels of staff.

Counseling on diet during pregnancy is a critical intervention to improve dietary intakes for pregnant women, which are suboptimal in LMICs, particularly India (31, 32). Recent literature has highlighted several constraints for women to adopt nutritious diets, including resource constraints leading to limited affordability and accessibility to nutritious foods, dependence on husbands to procure food, and food preferences (33, 34). Context-appropriate diet counseling is important to help women understand which locally available foods they should consume to provide adequate nutrition for themselves and their children. Counseling of husbands can also help to improve family support and build awareness of the importance of ensuring nutrition during pregnancy. Our study results indicated moderate levels of counseling on overall diet diversity and certain food groups, including green leafy vegetables, with gaps for counseling on other foods, particularly animal source foods. Closing these gaps is essential to improve quality of counseling and its effectiveness.

Although WHO (2016) (35) recommendations on maintaining healthy weight gain within a specified range are tailored to categories of prepregnancy BMI, current recommendations on calculating weight gain, counseling on weight gain, recordkeeping, and use of data are not specified by national protocols (14). Existing protocols do not specify weight-gain counseling based on BMI but recommend an average weight gain of 10–12 kg. In this study, gaps were prevalent in exposure to topics of weight gain during trainings and knowledge of optimal weight gain, leading to low provision of weight monitoring and weight-gain advice.

The secular trends observed could be explained by the parallel implementation of a national nutrition agenda and the systems-strengthening efforts adopted by A&T. The Indian government nationally prioritized the integration of maternal nutrition services into trainings for FLWs and community-based events focused on maternal nutrition. A&T's maternal nutrition interventions included technical assistance on supply chain and anemia management and sharing of behavior change materials across all districts in Uttar Pradesh; these activities may have led to some spillover effects.

In this study, data on training, knowledge, and service delivery were self-reported by FLWs, with potential for social desirability and recall bias. To objectively measure FLWs’ performance, we included direct observations of counseling to reduce potential bias from reported measures. The qualitative data collection was limited to 4 intervention blocks and may not reflect issues pertaining to nonintervention blocks or the larger study area. Finally, subjective measures used in qualitative interviews, including asking FLWs about their perceptions of supervision received, may have had response bias due to sociocultural norms that discourage criticism of supervisors. We reduced this bias by ensuring privacy during interviews and reassuring participants about response confidentiality.

To our knowledge, our study is one of the first to conduct an intervention-specific PIP analysis to examine the integration of a multifaceted maternal nutrition intervention into existing government systems, offering a unique contribution to the current literature. Many of the identified gaps in FLW knowledge, service delivery, counseling, and supply chains—and the reasons for the gaps—are likely experienced in similar programs in other contexts. The method used allowed going beyond overall impact-focused analyses (which have mostly explained successes rather than limited impacts of interventions) and can be used in analogous implementation research elsewhere. Systems-strengthening efforts within complex programs must focus on the specific demands of each intervention, which can be elucidated by conducting an intervention-specific PIP analysis to understand the areas of successes and challenges unique to different interventions.

## Supplementary Material

nxab390_Supplemental_File

## Notes

Supported by the Bill & Melinda Gates Foundation, through Alive & Thrive, managed by FHI Solutions, and the CGIAR Research Program on Agriculture for Nutrition and Health (A4NH), led by the International Food Policy Research Institute.

Author disclosures: SG, TF, JE-A, and PKS were members of the program implementation team that designed and implemented the interventions studied and reported on in this manuscript. They reviewed the manuscript and provided contextual interpretation of the results, but final decisions for manuscript content lay with the primary authors from the evaluation team (SK, PHN, LMT, EAF, and PM). All other authors report no conflicts of interest.

Supplemental Tables 1–5 are available from the “Supplementary data” link in the online posting of the article and from the same link in the online table of contents at https://academic.oup.com/jn/.

Abbreviations used: A&T, Alive & Thrive; ANC, antenatal care; ANM, auxiliary nurse midwife; ASHA, accredited social health activist; AWW, Anganwadi worker; DID, difference-in-differences; FLW, frontline worker; I-ANC, intensive antenatal care; ICDS, Integrated Child Development Services; LMIC, low- and middle-income country; PIP, program impact pathway; pp, percentage points; S-ANC, standard antenatal care; VHSND, village health, sanitation, and nutrition day.

## References

[1] Victora CG, Christian P, Vidaletti LP, Gatica-Dominguez G, Menon P, Black RE. Revisiting maternal and child undernutrition in low-income and middle-income countries: variable progress towards an unfinished agenda. Lancet North Am Ed. 2021;397(10282):1388–99. 10.1016/S0140-6736(21)00394-9 PMC7613170 33691094

[2] Black RE, Victora CG, Walker SP, Bhutta ZA, Christian P, de Onis M, Ezzati M, Grantham-McGregor S, Katz J, Martorell R et al. Maternal and child undernutrition and overweight in low-income and middle-income countries. Lancet North Am Ed. 2013;382(9890):427–51. 10.1016/S0140-6736(13)60937-X 23746772

[3] Black RE, Allen LH, Bhutta ZA, Caulfield LE, de Onis M, Ezzati M, Mathers C, Rivera J; Maternal, Child Undernutrition Study Group. Maternal and child undernutrition: global and regional exposures and health consequences. Lancet North Am Ed. 2008;371(9608):243–60. 10.1016/S0140-6736(07)61690-0 18207566

[4] India State-Level Disease Burden Initiative Malnutrition Collaborators . The burden of child and maternal malnutrition and trends in its indicators in the states of India: the Global Burden of Disease Study 1990-2017. Lancet Child Adolesc Health. 2019;3(12):855–70. 31542357 10.1016/S2352-4642(19)30273-1 PMC6839043

[5] International Institute for Population Sciences, ICF .National Family Health Survey (NFHS-4), 2015-16: India. Mumbai (India): IIPS; 2017.;

[6] Victora CG, Adair L, Fall C, Hallal PC, Martorell R, Richter L, Sachdev HS; Maternal, Child Undernutrition Study Group. Maternal and child undernutrition: consequences for adult health and human capital. Lancet North Am Ed. 2008;371(9609):340–57. 10.1016/S0140-6736(07)61692-4 PMC2258311 18206223

[7] Chou VB, Walker N, Kanyangarara M. Estimating the global impact of poor quality of care on maternal and neonatal outcomes in 81 low- and middle-income countries: a modeling study. PLoS Med. 2019;16(12):e1002990. 31851685 10.1371/journal.pmed.1002990 PMC6919595

[8] Heidkamp RA, Wilson E, Menon P, Kuo H, Walton S, Gatica-Dominguez G, Crochemore da Silva I, Aung T, Hajeebhoy N, Piwoz E. How can we realise the full potential of health systems for nutrition?. BMJ. 2020;368:l6911. 31983682 10.1136/bmj.l6911 PMC7461910

[9] Ramakrishnan U, Imhoff-Kunsch B, Martorell R. Maternal nutrition interventions to improve maternal, newborn, and child health outcomes. Nestle Nutr Inst Workshop Ser. 2014;78:71–80. 24504208 10.1159/000354942

[10] Victora CG, Barros FC, Assuncao MC, Restrepo-Mendez MC, Matijasevich A, Martorell R. Scaling up maternal nutrition programs to improve birth outcomes: a review of implementation issues. Food Nutr Bull. 2012;33(2, Suppl 1):S6–S26. 22913105 10.1177/15648265120332S102

[11] Torlesse H, Benedict RK, Craig HC, Stoltzfus RJ. The quality of maternal nutrition and infant feeding counselling during antenatal care in South Asia. Matern Child Nutr. 2021;17(3):e13153. 33554434 10.1111/mcn.13153 PMC8189234

[12] Nguyen PH, Avula R, Tran LM, Sethi V, Kumar A, Baswal D, Hajeebhoy N, Ranjan A, Menon P. Missed opportunities for delivering nutrition interventions in first 1000 days of life in India: insights from the National Family Health Survey, 2006 and 2016. BMJ Global Health. 2021;6(2):e003717. 10.1136/bmjgh-2020-003717 PMC7908280 33627359

[13] POSHAN Abhiyaan . PM's overarching scheme for holistic nourishment. [Internet] [cited June 2021]. Available from: http://poshanabhiyaan.gov.in/#/.

[14] Ministry of Health and Family Welfare . National guidelines on antenatal care for ANMs and medical officers government of India. Government of India:New Delhi, India; 2020.

[15] Heidkamp RA, Piwoz E, Gillespie S, Keats EC, D'Alimonte MR, Menon P, Das JK, Flory A, Clift JW, Ruel MT et al. Mobilising evidence, data, and resources to achieve global maternal and child undernutrition targets and the Sustainable Development Goals: an agenda for action. Lancet North Am Ed. 2021;397(10282):1400–18. 10.1016/S0140-6736(21)00568-7 33691095

[16] Nguyen PH, Kachwaha S, Tran LM, Avula R, Young MF, Ghosh S, Sharma PK, Escobar-Alegria J, Forissier T, Patil S et al. Strengthening nutrition interventions in antenatal care services affects dietary intake, micronutrient intake, gestational weight gain, and breastfeeding in Uttar Pradesh, India: results of a cluster-randomized program evaluation. J Nutr. 2021;151(8):2282–95. 34038529 10.1093/jn/nxab131 PMC8349122

[17] Pullar J, Wickramasinghe K, Demaio AR, Roberts N, Perez-Blanco KM, Noonan K, Townsend N. The impact of maternal nutrition on offspring's risk of non-communicable diseases in adulthood: a systematic review. J Global Health. 2019;9(2):020405. 10.7189/jogh.09.020405 PMC6790233 31656604

[18] Rawat R, Nguyen PH, Ali D, Saha K, Alayon S, Kim SS, Ruel M, Menon P. Learning how programs achieve their impact: embedding theory-driven process evaluation and other program learning mechanisms in Alive & Thrive. Food Nutr Bull. 2013;34:S212–25. 24261078 10.1177/15648265130343S207

[19] Avula R, Menon P, Saha KK, Bhuiyan MI, Chowdhury AS, Siraj S, Haque R, Jalal CS, Afsana K, Frongillo EA. A program impact pathway analysis identifies critical steps in the implementation and utilization of a behavior change communication intervention promoting infant and child feeding practices in Bangladesh. J Nutr. 2013;143(12):2029–37. 24068790 10.3945/jn.113.179085

[20] Kim SS, Ali D, Kennedy A, Tesfaye R, Tadesse AW, Abrha TH, Rawat R, Menon P. Assessing implementation fidelity of a community-based infant and young child feeding intervention in Ethiopia identifies delivery challenges that limit reach to communities: a mixed-method process evaluation study. BMC Public Health. 2015;15(1):316. 25879417 10.1186/s12889-015-1650-4 PMC4392481

[21] Nguyen PH, Menon P, Keithly SC, Kim SS, Hajeebhoy N, Tran LM, Ruel MT, Rawat R. Program impact pathway analysis of a social franchise model shows potential to improve infant and young child feeding practices in Vietnam. J Nutr. 2014;144(10):1627–36. 25143372 10.3945/jn.114.194464

[22] Alive & Thrive: IPE Global Limited India: final report: integrating maternal nutrition interventions in existing government MNCH services in two districts of Uttar Pradesh: Alive & Thrive India. Alive & Thrive: New Delhi, India. 2019.

[23] Zoellner J, Harris JE. Mixed-methods research in nutrition and dietetics. J Acad Nutr Diet. 2017;117(5):683–97. 28284525 10.1016/j.jand.2017.01.018

[24] Leech NL, Onwuegbuzie AJ. A typology of mixed methods research designs. Quality Quantity. 2009;43:265–75.

[25] Gertler P, Martinez S, Premand P, Rawlings L, Vermeersch C. Impact evaluation in practice. Washington (DC): World Bank Publications; 2011.

[26] Richards L . Handling qualitative data. London (UK): SAGE; 2006.;

[27] Young MF, Bootwala A, Kachwaha S, Avula R, Ghosh S, Sharma PK, Shastri VD, Forissier T, Menon P, Nguyen PH. Understanding implementation and improving nutrition interventions: barriers and facilitators of using data strategically to inform the implementation of maternal nutrition in Uttar Pradesh, India. Curr Dev Nutr. 2021;5(6):nzab081. 34222761 10.1093/cdn/nzab081 PMC8242137

[28] Patton M . Qualitative research and evaluation methods. 4th ed. Thousand Oaks (CA): SAGE; 2002.

[29] Kok MC, Dieleman M, Taegtmeyer M, Broerse JE, Kane SS, Ormel H, Tijm MM, de Koning KA. Which intervention design factors influence performance of community health workers in low- and middle-income countries? A systematic review. Health Policy Plan. 2015;30(9):1207–27. 25500559 10.1093/heapol/czu126 PMC4597042

[30] Omotayo MO, Dickin KL, Pelletier DL, Martin SL, Kung'u JK, Stoltzfus RJ. Feasibility of integrating calcium and iron-folate supplementation to prevent preeclampsia and anemia in pregnancy in primary healthcare facilities in Kenya. Matern Child Nutr. 2018;14(Suppl 1):e12437. 10.1111/mcn.12437 PMC6866141 29493897

[31] GBD 2017 Diet Collaborators . Health effects of dietary risks in 195 countries, 1990-2017: a systematic analysis for the Global Burden of Disease Study 2017. Lancet North Am Ed. 2019;393(10184):1958–72. 10.1016/S0140-6736(19)30041-8 PMC6899507 30954305

[32] Lee SE, Talegawkar SA, Merialdi M, Caulfield LE. Dietary intakes of women during pregnancy in low- and middle-income countries. Public Health Nutr. 2013;16(8):1340–53. 23046556 10.1017/S1368980012004417 PMC10271363

[33] Raghunathan K, Headey D, Herforth A. Affordability of nutritious diets in rural India. Food Policy. 2021;99:101982. 33746340 10.1016/j.foodpol.2020.101982 PMC7957322

[34] Kachwaha S, Nguyen PH, DeFreese M, Avula R, Cyriac S, Girard A, Menon P. Assessing the economic feasibility of assuring nutritionally adequate diets for vulnerable populations in Uttar Pradesh, India: findings from a “Cost of the diet” analysis. Curr Dev Nutr. 2020;4(12):nzaa169. 33313474 10.1093/cdn/nzaa169 PMC7721462

[35] WHO . WHO recommendations on antenatal care for a positive pregnancy experience. Geneva (Switzerland): WHO; 2016. 28079998

