# Supplementary material for: Specificity Matters: Unpacking Impact Pathways of Individual Interventions within Bundled Packages Helps Interpret the Limited Impacts of a Maternal Nutrition Intervention in India
Source: J Nutr. 2021 Nov 18;152(2):612–29. doi: 10.1093/jn/nxab390 (PMC8826931; doi:10.1093/jn/nxab390)
Supplement: nxab390_Supplemental_File [file nxab390_supplemental_file.docx]

**Supplemental Table 1: Characteristics of frontline workers at baseline^1^**

|  | **AWW** | | **ASHA** | | **ANM** | |
| --- | --- | --- | --- | --- | --- | --- |
|  | **I-ANC area** | **S-ANC area** | **I-ANC area** | **S-ANC area** | **I-ANC area** | **S-ANC area** |
|  | ***n*=91** | ***n*=87** | ***n*=90** | ***n*=90** | ***n*=71** | ***n*=68** |
| Age of FLW | 43.0 ± 7.6 | 43.5 ± 7.4 | 37.5 ± 6.7 | 37.0 ± 6.5 | 45.8 ± 11.5 | 43.1 ± 11.7 |
| Highest class completed^2^ |  |  |  |  |  |  |
| Lower than primary | -- | -- | 0.0 | 2.2 | -- | -- |
| Lower than secondary | 1.1 | 3.4 | 46.7 | 48.9 | -- | -- |
| Secondary school | 40.7 | 55.2 | 45.6 | 37.8 | 52.1 | 32.4 |
| Graduate and above | 58.2 | 41.4 | 7.8 | 11.1 | 47.9 | 67.6 |
| Religion |  |  |  |  |  |  |
| Hindu | 98.9 | 97.7 | 100.0 | 97.8 | 94.4 | 95.6 |
| Muslim/ others | 1.1 | 2.3 | 0.0 | 2.2 | 5.6 | 4.4 |
| Caste category^3^ |  |  |  |  |  |  |
| Scheduled caste/tribe/other backward classes | 70.3 | 67.8 | 82.0 | 75.6 | 65.7 | 66.2 |
| General | 29.7 | 32.2 | 18.0 | 24.4 | 34.3 | 33.8 |
| Having other work with compensation received | 11.0 | 14.9 | 10.0 | 10.0 | 5.6 | 0.0 |

^1^Values are means ± SDs or percentages; ^2^Highest class completed: Lower than primary ≤class 5, Lower than secondary ≤class 9, Secondary school= classes 9-12, graduate and above= graduate/post-graduate education

^3^Among those who report caste category

ANM: Auxiliary Nurse Midwife; ASHA: Accredited Social Health Activist; AWW: Anganwadi Worker; FLW: Frontline worker; I-ANC: Intensive antenatal care; S-ANC: Standard antenatal care.

Supplemental Table 2: Details of reported counseling on IFA, by intervention area and survey round^1^

|  | **Baseline 2017** | | **Endline 2019** | |
| --- | --- | --- | --- | --- |
|  | **I-ANC area** | **S-ANC area** | **I-ANC area** | **S-ANC area** |
| **AWW** | ***n*=91** | ***n*=87** | ***n*=87** | ***n*=91** |
| Advised PW to take IFA regularly (1 tablet/day) | 41.8 | 51.7 | 54.0 | 69.2*^2^ |
| Take 180 IFA tablets during pregnancy | 24.2 | 35.6 | 48.3 | 28.6** |
| Continue to take 1 tablet/day till 6 months postpartum | 18.7 | 19.5 | 32.2 | 28.6 |
| Take IFA with water or lemon water | 25.3 | 25.3 | 65.5 | 60.4 |
| Advised to take IFA at night before bedtime | 40.7 | 39.1 | 62.1 | 49.5 |
| IFA prevents anemia | 33.0 | 25.3** | 37.9 | 30.8 |
| IFA reduce risk of low birth weight baby | 12.1 | 13.8 | 28.7 | 22.0 |
| IFA reduce risk of maternal death due to hemorrhage | 9.9 | 17.2 | 18.4 | 19.8 |
| IFA ensures the best development of the child | 6.6 | 13.8* | 9.2 | 9.9 |
| IFA reduces complication during pregnancy and birth | 7.7 | 8.0 | 18.4 | 9.9 |
| Information related to side effects | 11.0 | 8.0 | 8.0 | 6.6 |
| Reminded woman not to take IFA with tea or milk | 12.1 | 21.8 | 40.2 | 34.1 |
| Do not take IFA and calcium together | 4.4 | 10.3* | 24.1 | 27.5 |
| Do not take IFA tablet on an empty stomach | 17.6 | 19.5 | 28.7 | 20.9 |
| How PW can remind herself to take IFA | 14.3 | 17.2 | 35.6 | 23.1 |
| **ASHA** | ***n*=90** | ***n*=90** | ***n*=86** | ***n*=88** |
| Advised PW to take IFA regularly (1 tablet/day) | 64.4 | 58.9 | 60.5 | 67.0 |
| Take 180 IFA tablets during pregnancy | 33.3 | 42.2 | 52.3 | 33.0** |
| Continue to take 1 tablet/day till 6 months postpartum | 13.3 | 22.2 | 33.7 | 33.0 |
| Take IFA with water or lemon water | 30.0 | 26.7 | 64.0 | 53.4 |
| Advised to take IFA at night before bedtime | 48.9 | 44.4 | 46.5 | 47.7 |
| IFA prevents anemia | 28.9 | 33.3 | 32.6 | 30.7 |
| IFA reduce risk of low birth weight baby | 14.4 | 17.8 | 20.9 | 19.3 |
| IFA reduce risk of maternal death due to hemorrhage | 10.0 | 15.6 | 23.3 | 20.5 |
| IFA ensures the best development of the child | 12.2 | 12.2 | 15.1 | 13.6 |
| IFA reduces complication during pregnancy and birth | 15.6 | 16.7 | 20.9 | 17.0 |
| Information related to side effects | 11.1 | 11.1 | 5.8 | 5.7 |
| Reminded woman not to take IFA with tea or milk | 20.0 | 24.4 | 43.0 | 34.1 |
| Do not take IFA and calcium together | 11.1 | 17.8 | 33.7 | 26.1 |
| Do not take IFA tablet on an empty stomach | 21.1 | 22.2 | 23.3 | 28.4 |
| How PW can remind herself to take IFA | 12.2 | 13.3 | 36.0 | 20.5* |
| **ANM** | ***n*=71** | ***n*=68** | ***n*=58** | ***n*=69** |
| Advised PW to take IFA regularly (1 tablet/day) | 67.6 | 51.5 | 58.6 | 69.6 |
| Take 180 IFA tablets during pregnancy | 40.8 | 51.5 | 63.8 | 52.2 |
| Continue to take 1 tablet/day till 6 months postpartum | 22.5 | 30.9 | 32.8 | 29.0 |
| Take IFA with water or lemon water | 23.9 | 27.9 | 60.3 | 56.5 |
| Advised to take IFA at night before bedtime | 59.2 | 52.9 | 39.7 | 40.6 |
| IFA prevents anemia | 25.4 | 41.2 | 27.6 | 24.6** |
| IFA reduce risk of low birth weight baby | 18.3 | 25.0 | 48.3 | 20.3 |
| IFA reduce risk of maternal death due to hemorrhage | 21.1 | 11.8* | 27.6 | 20.3*** |
| IFA ensures the best development of the child | 16.9 | 17.6 | 17.2 | 15.9 |
| IFA reduces complication during pregnancy and birth | 11.3 | 13.2 | 15.5 | 11.6 |
| Information related to side effects | 7.0 | 13.2 | 6.9 | 8.7 |
| Reminded woman not to take IFA with tea or milk |  |  |  |  |
| Do not take IFA and calcium together | 19.7 | 22.1 | 44.8 | 21.7* |
| Do not take IFA tablet on an empty stomach | 7.0 | 11.8 | 31.0 | 29.0 |
| How PW can remind herself to take IFA | 25.4 | 25.0 | 36.2 | 13.0* |

^1^Values are percentages; ^2^Asterisks indicate different from I-ANC area at that time: *P< 0.05, ** P < 0.01, *** P < 0.001.

ANM: Auxiliary Nurse Midwife; ASHA: Accredited Social Health Activist; AWW: Anganwadi Worker; I-ANC: Intensive antenatal care; IFA: Iron and folic acid; PW: Pregnant women; S-ANC: Standard antenatal care.

Supplemental Table 3: Details of reported counseling on calcium, by intervention area and survey round^1^

|  | **Baseline 2017** | | **Endline 2019** | |
| --- | --- | --- | --- | --- |
|  | **I-ANC area** | **S-ANC area** | **I-ANC area** | **S-ANC area** |
| **AWW** | ***n*=91** | ***n*=87** | ***n*=87** | ***n*=91** |
| Advised PW to take calcium regularly (2 tablet/day) | 9.9 | 8.0 | 58.6 | 51.6 |
| Take 360 calcium tablets during pregnancy | 8.8 | 8.0 | 34.5 | 25.3 |
| Continue to take 2 tablets/day till 6 months postpartum | 8.8 | 9.2 | 32.2 | 25.3 |
| Do not take IFA and calcium together | 5.5 | 8.0 | 48.3 | 39.6 |
| Do not take calcium tablet on an empty stomach | 8.8 | 4.6 | 51.7 | 38.5 |
| Take the first calcium tablet after breakfast and the second tablet with lunch | 11.0 | 6.9 | 46.0 | 29.7*^2^ |
| Calcium helps in the development of bone and teeth | 18.7 | 9.2 | 54.0 | 41.8 |
| Calcium reduce risk of high blood pressure, swelling of body, with headache, nausea & vomiting and blurring of vision & fits | 6.6 | 9.2 | 41.4 | 28.6 |
| How to remind herself or have family member’s support to take one tablet daily | 2.2 | 6.9 | 5.7 | 6.6 |
| **ASHA** | ***n*=90** | ***n*=90** | ***n*=86** | ***n*=88** |
| Advised PW to take calcium regularly (2 tablet/day) | 8.9 | 14.4 | 62.8 | 58.0 |
| Take 360 calcium tablets during pregnancy | 8.9 | 10.0 | 37.2 | 31.8 |
| Continue to take 2 tablets/day till 6 months postpartum | 6.7 | 6.7 | 45.3 | 21.6*** |
| Do not take IFA and calcium together | 4.4 | 6.7 | 52.3 | 35.2* |
| Do not take calcium tablet on an empty stomach | 7.8 | 10.0 | 47.7 | 38.6 |
| Take the first calcium tablet after breakfast and the second tablet with lunch | 11.1 | 11.1 | 47.7 | 39.8 |
| Calcium helps in the development of bone and teeth | 11.1 | 14.4 | 60.5 | 50.0 |
| Calcium reduce risk of high blood pressure, swelling of body, with headache, nausea & vomiting and blurring of vision & fits | 8.9 | 10.0 | 33.7 | 23.9 |
| How to remind herself or have family member’s support to take one tablet daily | 11.1 | 4.4 | 8.1 | 6.8 |
| **ANM** | ***n*=71** | ***n*=68** | ***n*=58** | ***n*=69** |
| Advised PW to take calcium regularly (2 tablet/day) | 12.7 | 22.1 | 67.2 | 42.0** |
| Take 360 calcium tablets during pregnancy | 8.5 | 7.4 | 50.0 | 27.5* |
| Continue to take 2 tablets/day till 6 months postpartum | 8.5 | 17.6 | 41.4 | 23.2* |
| Do not take IFA and calcium together | 11.3 | 11.8 | 39.7 | 42.0 |
| Do not take calcium tablet on an empty stomach | 7.0 | 14.7 | 39.7 | 36.2 |
| Take the first calcium tablet after breakfast and the second tablet with lunch | 12.7 | 13.2 | 44.8 | 24.6* |
| Calcium helps in the development of bone and teeth | 16.9 | 22.1 | 70.7 | 52.2* |
| Calcium reduce risk of high blood pressure, swelling of body, with headache, nausea & vomiting and blurring of vision & fits | 12.7 | 10.3 | 20.7 | 20.3 |
| How to remind herself or have family member’s support to take one tablet daily | 5.6 | 8.8 | 0.0 | 7.2* |

^1^Values are percentages; ^2^Asterisks indicate different from I-ANC area at that time: *P< 0.05, ** P < 0.01, *** P < 0.001

ANM: Auxiliary Nurse Midwife; ASHA: Accredited Social Health Activist; AWW: Anganwadi Worker; I-ANC: Intensive antenatal care; IFA: Iron and folic acid; PW: Pregnant women; S-ANC: Standard antenatal care.

Supplemental Table 4: Details of reported counseling on diet diversity, by intervention area and survey round^1^

|  | **Baseline 2017** | | **Endline 2019** | |
| --- | --- | --- | --- | --- |
|  | **I-ANC area** | **S-ANC area** | **I-ANC area** | **S-ANC area** |
|  | **Percent** | **Percent** | **Percent** | **Percent** |
| **AWW** | ***n*=91** | ***n*=87** | ***n*=87** | ***n*=91** |
| PW to eat five different types of food in addition to roti/rice everyday | 59.3 | 57.5 | 74.7 | 69.2 |
| PW have to eat dark green leafy vegetable everyday | 84.6 | 87.4 | 81.6 | 83.5 |
| PW have to eat yellow/orange fruits or vegetables everyday | 65.9 | 60.9 | 77 | 72.5 |
| PW have to eat thick daal everyday | 58.2 | 60.9 | 65.5 | 63.7 |
| PW have to eat fish/meat everyday | 33.0 | 35.6 | 64.4 | 41.8**^2^ |
| PW have to eat an egg everyday, if acceptable | 28.6 | 27.6 | 52.9 | 41.8 |
| PW have to eat milk/milk products everyday | 42.9 | 43.7 | 49.4 | 48.4 |
| **ASHA** | ***n*=90** | ***n*=90** | ***n*=86** | ***n*=88** |
| PW to eat five different types of food in addition to roti/rice everyday | 67.8 | 54.4 | 69.8 | 68.2 |
| PW have to eat dark green leafy vegetable everyday | 82.2 | 88.9 | 84.9 | 89.8 |
| PW have to eat yellow/orange fruits or vegetables everyday | 58.9 | 53.3 | 57.0 | 78.4 |
| PW have to eat thick daal everyday | 64.4 | 68.9 | 61.6 | 59.1 |
| PW have to eat fish/meat everyday | 32.2 | 32.2 | 60.5 | 53.4 |
| PW have to eat an egg everyday, if acceptable | 34.4 | 33.3 | 45.3 | 43.2 |
| PW have to eat milk/milk products everyday | 38.9 | 56.7 | 53.5 | 61.4 |
| **ANM** | ***n*=71** | ***n*=68** | ***n*=58** | ***n*=69** |
| PW to eat five different types of food in addition to roti/rice everyday | 54.9 | 60.3 | 48.3 | 44.9 |
| PW have to eat dark green leafy vegetable everyday | 80.3 | 89.7 | 82.8 | 89.9 |
| PW have to eat yellow/orange fruits or vegetables everyday | 57.7 | 67.6 | 63.8 | 66.7 |
| PW have to eat thick daal everyday | 62.0 | 58.8 | 65.5 | 56.5 |
| PW have to eat fish/meat everyday | 35.2 | 38.2 | 43.1 | 47.8 |
| PW have to eat an egg everyday, if acceptable | 38.0 | 33.8 | 48.3 | 27.5* |
| PW have to eat milk/milk products everyday | 39.4 | 54.4 | 50.0 | 47.8 |

^1^Values are percentages; ^2^Asterisks indicate different from I-ANC area at that time: *P< 0.05, ** P < 0.01, *** P < 0.001

ANM: Auxiliary Nurse Midwife; ASHA: Accredited Social Health Activist; AWW: Anganwadi Worker; I-ANC: Intensive antenatal care; S-ANC: Standard antenatal care.

Supplemental Table 5: Details of reported counseling on weight gain monitoring, by intervention area and survey round^1^

|  | **Baseline 2017** | | **Endline 2019** | |
| --- | --- | --- | --- | --- |
|  | **I-ANC area** | **S-ANC area** | **I-ANC area** | **S-ANC area** |
|  | **Percent** | **Percent** | **Percent** | **Percent** |
| **AWW** | ***n*=91** | ***n*=87** | ***n*=87** | ***n*=91** |
| Weigh monthly during pregnancy | 47.3 | 59.8 | 52.9 | 64.8 |
| Attend VHSND to record weight | 20.9 | 29.9 | 36.8 | 39.6 |
| Record weight in Mother and child protection card | 22.0 | 35.6 | 31 | 24.2 |
| Women should gain 1.5-2 kg/month from the fourth month of pregnancy | 22.0 | 29.9 | 59.8 | 40.7*^2^ |
| A woman should gain 10-12 kg weight during pregnancy | 29.7 | 37.9 | 41.4 | 34.1 |
| Gaining weight indicates proper growth of the fetus | 27.5 | 41.4 | 33.3 | 27.5 |
| **ASHA** | ***n*=90** | ***n*=90** | ***n*=86** | ***n*=88** |
| Weigh monthly during pregnancy | 38.9 | 46.7 | 55.8 | 52.3 |
| Attend VHSND to record weight | 27.8 | 31.1 | 59.3 | 37.5** |
| Record weight in Mother and child protection card | 28.9 | 31.1 | 30.2 | 30.7 |
| Women should gain 1.5-2 kg/month from the fourth month of pregnancy | 22.2 | 30.0 | 54.7 | 36.4* |
| A woman should gain 10-12 kg weight during pregnancy | 31.1 | 28.9 | 41.9 | 36.4 |
| Gaining weight indicates proper growth of the fetus | 14.4 | 23.3 | 16.3 | 35.2** |
| **ANM** | ***n*=71** | ***n*=68** | ***n*=58** | ***n*=69** |
| Weigh monthly during pregnancy | 49.3 | 61.8 | 58.6 | 52.2 |
| Attend VHSND to record weight | 23.9 | 36.8 | 50.0 | 39.1 |
| Record weight in Mother and child protection card | 32.4 | 42.6 | 50.0 | 27.5* |
| Women should gain 1.5-2 kg/month from the fourth month of pregnancy | 33.8 | 52.9 | 60.3 | 37.7** |
| A woman should gain 10-12 kg weight during pregnancy | 31.0 | 44.1 | 43.1 | 29.0 |
| Gaining weight indicates proper growth of the fetus | 25.4 | 35.3 | 22.4 | 21.7 |

^1^Values are percentages; ^2^Asterisks indicate different from I-ANC area at that time: *P< 0.05, ** P < 0.01, *** P < 0.001.

ANM: Auxiliary Nurse Midwife; ASHA: Accredited Social Health Activist; AWW: Anganwadi Worker; I-ANC: Intensive antenatal care; S-ANC: Standard antenatal care; VHSND: Village Health, Sanitation and Nutrition Day;
